# Novel duck reovirus σC hijacks the mitochondrial COQ6–CoQ10 axis to drive NLRP3-dependent pyroptosis

**DOI:** 10.1371/journal.ppat.1014392

**Published:** 2026-07-07

**Authors:** Hui Yan, Lei Bei, Mingrui Zhao, Jiajun Wang, Pengxiang Jin, Chunmei Xu, Lin Ju, Yu Meng, Shijin Jiang, Ruihua Zhang

**Affiliations:** 1 Department of Preventive Veterinary Medicine, College of Veterinary Medicine, Shandong Agricultural University, Tai’an, China; 2 Shandong Provincial Key Laboratory of Zoonoses, Shandong Agricultural University, Tai’an, China; University of Arkansas for Medical Sciences, UNITED STATES OF AMERICA

## Abstract

Mitochondrial metabolic homeostasis serves as a critical checkpoint that integrates cellular bioenergetics with innate immunity. While viruses often subvert mitochondrial functions to manipulate cell fate, the specific viral determinants that directly reprogram host metabolic enzymes to drive inflammatory injury remain incompletely defined. Here, using infection with a highly virulent novel duck reovirus (NDRV) strain as a model of systemic immunopathology, we identify the viral capsid protein σC as a metabolic virulence factor. We show that σC binds to the mitochondrial targeting sequence (MTS) of COQ6, a conserved monooxygenase required for coenzyme Q10 (CoQ10) biosynthesis, and blocks its mitochondrial import, thereby disrupting CoQ10 homeostasis. This metabolic blockade induces mitochondrial dysfunction, characterized by mtROS accumulation and the cytosolic release of oxidized mitochondrial DNA (ox-mtDNA). We demonstrate that ox-mtDNA acts as a danger signal that triggers NLRP3 activation and downstream gasdermin E (GSDME)-dependent pyroptosis. Moreover, σC knockdown in infected cells reduces ox-mtDNA release and attenuates pyroptosis. CoQ10 supplementation restores mitochondrial homeostasis and alleviates NDRV-induced inflammatory pathology *in vitro* and *in vivo* without detectably reducing viral RNA loads, consistent with a host-directed disease tolerance mechanism. Collectively, these findings define a σC–COQ6–CoQ10–ox-mtDNA–NLRP3 axis that links a structural avian orthoreovirus protein to metabolic reprogramming and highlight the COQ6–CoQ10 pathway as a tractable therapeutic target for limiting virus-induced inflammatory tissue damage.

## Author summary

Viral manipulation of host metabolism is a key driver of pathogenesis, yet the specific viral determinants that reprogram metabolic enzymes to ignite inflammation remain poorly understood. In this study, we used infection with novel duck reovirus (NDRV), which causes severe splenic necrosis in ducklings, as a model to identify the viral capsid protein σC as a metabolic virulence factor. We show that σC physically binds to the host enzyme COQ6 and blocks its mitochondrial import, thereby impairing the biosynthesis of coenzyme Q10 (CoQ10). This metabolic blockade triggers mitochondrial dysfunction and the cytosolic release of oxidized mitochondrial DNA (ox-mtDNA), which acts as a danger signal to activate the NLRP3 inflammasome and drive pyroptosis. Therapeutic supplementation with CoQ10 restores mitochondrial homeostasis and mitigates tissue injury without detectably reducing viral RNA loads, consistent with a host-directed disease tolerance mechanism. Our findings reveal a direct axis linking a viral protein to metabolic reprogramming and immunopathology, highlighting mitochondrial redox maintenance as a viable strategy to limit virus-induced tissue damage.

## Introduction

Mitochondria function as a critical checkpoint integrating cellular bioenergetics with innate immunity [1–3]. Central to this integration is the mitochondrial electron transport chain (ETC), which maintains the proton gradient required for ATP synthesis. This bioenergetic balance is frequently subverted by pathogens to manipulate cell fate [4]. Viral infection can disrupt ETC dynamics, leading to the accumulation of mitochondrial reactive oxygen species (mtROS), dissipation of mitochondrial membrane potential (ΔΨ_m_), and the release of danger-associated molecular patterns. Such bioenergetic stress is not merely a metabolic consequence but also acts as a potent amplifier of inflammation. Notably, cytosolic oxidized mitochondrial DNA (ox-mtDNA) has emerged as a key danger signal that engages the NLRP3 inflammasome, linking mitochondrial stress to caspase-1 (CASP-1) activation and inflammatory cell death [5–8]. Although mitochondrial dysfunction is a known driver of inflammasome activation in mammalian models [9–12], the specific mechanisms by which avian viral pathogens directly reprogram host metabolic enzymes to initiate the ox-mtDNA–NLRP3 cascade remain poorly defined.

Avian orthoreoviruses (ARVs; species *Orthoreovirus avis*) provide a valuable model for investigating these mechanisms. Among clinically relevant isolates, the novel duck reovirus (NDRV) genotype exhibits markedly greater virulence than classical Muscovy duck reovirus (MDRV), causing severe splenic necrosis and extensive immune cell depletion in ducks [13–17]. This systemic immunopathology offers a tractable system for dissecting host-driven tissue injury. Furthermore, the avian host presents a streamlined gasdermin (GSDM) landscape. Unlike humans and mice, which encode multiple pore-forming GSDM paralogs [18–20], ducks primarily express GSDMA and GSDME and naturally lack GSDMD [21,22]. In mammalian contexts, the dominant role of GSDMD in canonical inflammasome signaling often masks the contribution of GSDME, complicating the dissection of its specific immunological function. Thus, this evolutionary context eliminates the confounding role of GSDMD, allowing more precise analysis of GSDME-dependent pyroptosis. Yet, the upstream viral triggers and mitochondrial signals that engage this pyroptosis axis during NDRV infection remain poorly understood.

The NDRV genome consists of ten double-stranded RNA (dsRNA) segments encoding eight structural proteins (σB, σC, μB, λA, λB, λC, μA, and σA) and four nonstructural proteins (μNS, σNS, P18, and P10) [23,24]. While innate sensing of genomic dsRNA is a well-established driver of antiviral inflammation, this RNA-centric view may overlook the ability of viral proteins to directly modulate host bioenergetics. The outer-capsid protein σC is canonically known for mediating cell attachment and determining serotype [25–27]. Although previous studies have primarily focused on its roles in viral entry and replication, whether reovirus proteins exploit host bioenergetics to drive pathogenesis remains unclear. Whether σC functions as a metabolic virulence factor that induces mitochondrial dysfunction and downstream immunopathology has not been explored. Defining such protein-driven mechanisms is essential for understanding how NDRV couples mitochondrial stress to pyroptotic tissue injury.

In this study, we identify σC as a metabolic virulence factor that links viral infection to pyroptotic tissue injury. We show that σC specifically targets the host enzyme COQ6, blocks its mitochondrial import and disrupts CoQ10 biosynthesis. This metabolic disruption triggers the accumulation of cytosolic ox-mtDNA, which subsequently activates the NLRP3–CASP-1–GSDME pathway. Importantly, restoring CoQ10 homeostasis alleviates splenic pathology without detectably reducing viral RNA loads, consistent with a host-directed disease-tolerance mechanism. Collectively, these findings define a σC–COQ6–CoQ10–ox-mtDNA–NLRP3 axis and reveal how a structural viral protein reprograms host bioenergetics to drive immunopathology.

## Results

### NDRV infection causes splenic inflammatory pathology and activates pyroptosis signaling in ducklings

**Fig 1:**
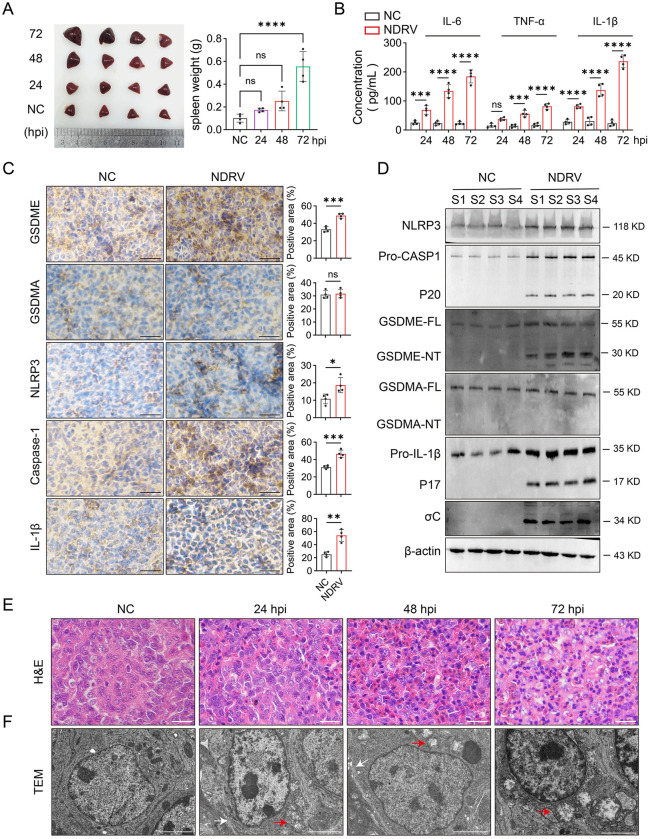
NDRV infection induces splenic pathology and activates inflammasome-and GSDME-associated signaling in ducklings. **(A)** Representative gross morphology of spleens from mock- and NDRV-infected ducklings at 24, 48 and 72 hpi (n = 4 per group). **(B)** Serum concentrations of IL-6, TNF-α, and IL-1β at the indicated time points post-infection (n = 4 per group). **(C)** IHC analysis of CASP-1, GSDME, GSDMA, NLRP3, and IL-1β in spleen sections at 48 hpi. Scale bar, 25 μm. **(D)** WB analysis of NLRP3, CASP-1, GSDME, GSDMA and IL-1β in spleens at 48 hpi, with β-actin as the loading control. NDRV infection was confirmed by the detection of the σC protein. **(E)** Representative H&E staining of spleen sections. Scale bar, 20 μm. **(F)** Representative TEM images showing ultrastructural changes in spleen tissues. Scale bar, 200 nm. Data are shown as the mean ± SD. Statistical analyses were performed using ANOVA followed by Tukey’s multiple-comparisons test. *p < 0.05, **p < 0.01, ***p < 0.001, ****p < 0.0001.

NDRV infection resulted in visible splenic enlargement and progressive gross lesions over time. Macroscopically, spleens from infected ducklings were friable and exhibited multifocal gray-white necrotic foci on the capsular surface (Fig 1A). Concurrently, serum concentrations of the inflammatory cytokines IL-6, TNF-α, and IL-1β began to rise at 24 hours post-infection (hpi) and increased progressively over time (Fig 1B), indicative of a systemic inflammatory response. Histopathological analysis further revealed marked disruption of the splenic architecture, characterized by blurred boundaries between the red and white pulp and prominent infiltration of inflammatory cells. As the infection advanced, splenic sinusoids progressively dilated and became engorged with erythrocytes (Fig 1E). Ultrastructural analysis using transmission electron microscopy (TEM) further confirmed severe tissue injury. With increasing infection duration, splenic cells displayed progressive cellular swelling and compromised membrane integrity. Mitochondria appeared reduced in number and showed pronounced swelling and deformation, along with diminished or absent cristae (Fig 1F).

We next assessed the *in vivo* engagement of inflammasome and GSDME-associated pathways. Immunohistochemistry (IHC) at 48 hpi demonstrated increased staining intensity for NLRP3, CASP-1, GSDME, GSDMA, IL-1β and NDRV within the infected spleens, with corresponding quantification analyses presented in Fig 1C and S1 Fig. Consistently, immunoblotting of splenic lysates at 48 hpi revealed increased levels of these markers, including the characteristic appearance of the cleaved CASP-1 p20 subunit, the GSDME-NT fragment (~30 kDa), and mature IL-1β. β-actin was used as a loading control. NDRV infection was independently verified by detecting the viral σC protein (Fig 1D). We also examined GSDMA activation following NDRV infection in DEFs. No obvious cleaved GSDMA fragment was detected within 48 hpi under our experimental conditions, and no marked activation-associated processing of full-length GSDMA was observed. Collectively, these data indicate that NDRV infection induces severe splenic inflammatory pathology in ducklings, which correlates with *in vivo* inflammasome activation and GSDME-mediated pyroptosis.

### NDRV infection induces pyroptotic features in PBMCs

**Fig 2:**
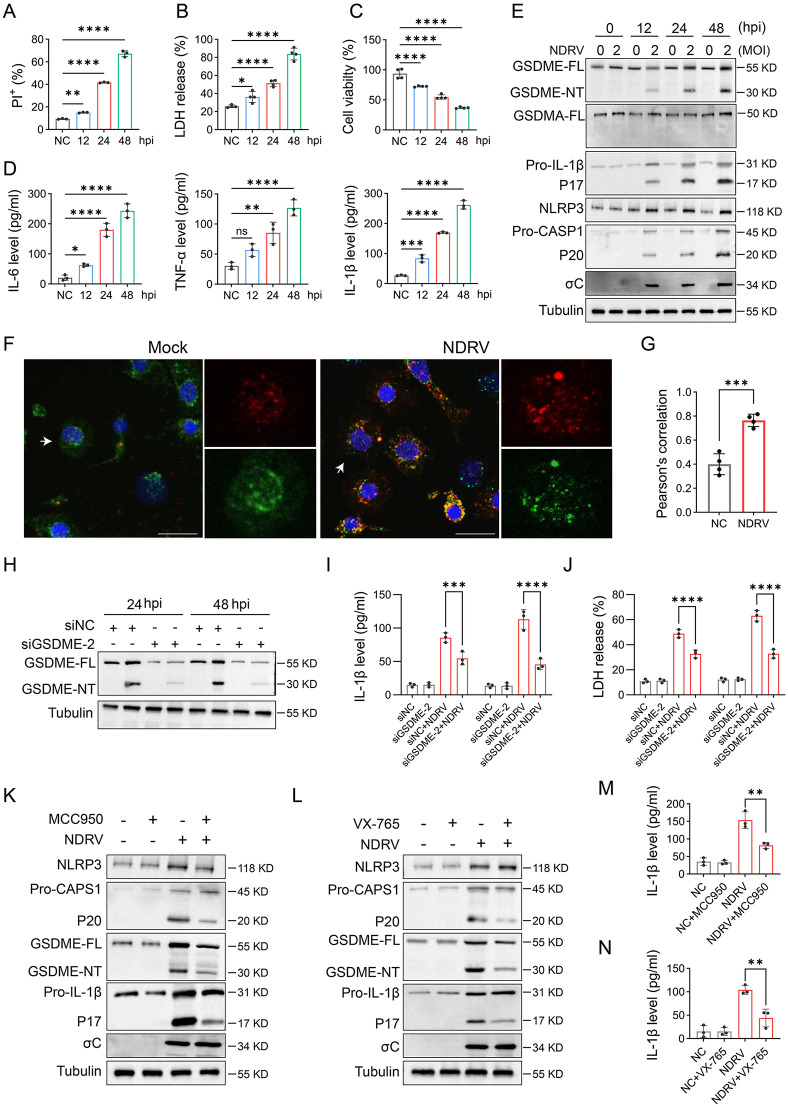
NDRV infection induces pyroptosis in PBMCs and activates NLRP3–CASP-1–GSDME signaling. **(A)** Quantification of PI-positive cells following NDRV infection at the indicated time points. **(B)** LDH release in culture supernatants from NDRV-infected PBMCs. **(C)** Cell viability of PBMCs measured using a CCK-8 assay following NDRV infection. **(D)** IL-6, TNF-α, and IL-1β concentrations in culture supernatants measured by ELISA. **(E)** WB analysis of GSDME, GSDMA, IL-1β, NLRP3, CASP-1 and the viral σC protein in NDRV-infected PBMCs. Tubulin was used as the loading control. **(F)** Confocal microscopy showing colocalization of ASC (red) and NLRP3 (green) in PBMCs at 24 hpi. Arrows indicate ASC-NLRP3 colocalization. Scale bar, 5 μm. **(G)** Quantification of ASC–NLRP3 colocalization by Pearson’s correlation coefficient. **(H)** WB analysis of GSDME expression and cleavage in NDRV-infected DEFs transfected with control siRNA or GSDME-targeting siRNA at 24 and 48 hpi. Tubulin was used as the loading control. **(I)** IL-1β concentrations in culture supernatants from NDRV-infected DEFs with or without GSDME knockdown. **(J)** LDH release from NDRV-infected DEFs with or without GSDME knockdown. **(K)** WB analysis of NLRP3, CASP-1, GSDME, IL-1β, and σC in NDRV-infected PBMCs with or without MCC950 treatment. Tubulin was used as the loading control. **(L)** WB analysis of NLRP3, CASP-1, GSDME, IL-1β, and σC in NDRV-infected PBMCs with or without VX-765 treatment. Tubulin was used as the loading control. **(M)** IL-1β concentrations in culture supernatants from NDRV-infected PBMCs with or without MCC950 treatment. **(N)** IL-1β concentrations in culture supernatants from NDRV-infected PBMCs with or without VX-765 treatment. Data are shown as mean ± SD values. Statistical significance was determined by ANOVA followed by Tukey’s multiple-comparisons test for multiple-group comparisons and an unpaired Student’s t-test for two-group comparisons. *p < 0.05, **p < 0.01, ***p < 0.001, ****p < 0.0001.

Given the prominent pyroptosis-associated features observed *in vivo*, we established an *in vitro* model using peripheral blood mononuclear cells (PBMCs) to investigate the underlying mechanisms. This model recapitulated the diverse immune cell populations found in the spleen. Following NDRV infection, PBMC viability declined significantly (Fig 2C), accompanied by increased propidium iodide (PI) uptake (Fig 2A) and elevated lactate dehydrogenase (LDH) release (Fig 2B), indicative of compromised cell membrane integrity. Notably, these cytotoxic changes became more pronounced in a time-dependent manner.

NDRV infection also promoted the production of inflammatory cytokines. ELISA measurements showed that IL-6, TNF-α, and IL-1β levels in culture supernatants increased significantly throughout the infection course (Fig 2D). Consistently, Western blot (WB) analysis detected upregulated expression of NLRP3, CASP-1, GSDME and IL-1β, together with the cleaved products CASP-1 p20 subunit, the GSDME-NT (~30 kDa) fragment, and mature IL-1β. Concurrent detection of the viral σC protein confirmed successful infection (Fig 2E). We also examined GSDMA activation under the same conditions, but no obvious GSDMA cleavage was detected within 48 hpi (Fig 2E). These data demonstrate that NDRV infection drives time-dependent inflammatory and pyroptotic responses in PBMCs.

### NDRV activates NLRP3 and CASP-1–dependent GSDME cleavage

To determine whether NDRV-induced pyroptosis in PBMCs is mediated by the NLRP3 inflammasome, we assessed inflammasome complex assembly. Confocal microscopy at 24 hpi showed clear colocalization of the adaptor protein ASC with NLRP3, confirming the formation of ASC-NLRP3 inflammasome specks (Fig 2F). Quantitative colocalization analysis further confirmed increased ASC–NLRP3 colocalization following NDRV infection (Fig 2G). To functionally confirm the involvement of this pathway, PBMCs were pretreated with the NLRP3 inhibitor MCC950 or the CASP-1 inhibitor VX-765 prior to NDRV infection. Both inhibitors markedly reduced IL-1β and LDH secretion (Fig 2M, 2N, S2C, and S2D Fig). Consistently, WB analysis confirmed that MCC950 and VX-765 dampened inflammasome signaling, resulting in reduced downstream GSDME processing and IL-1β maturation (Fig 2K and 2L). Together, these results indicate that NDRV infection activates the NLRP3 inflammasome in PBMCs, engaging a CASP-1-dependent pathway that is crucial for pyroptosis execution.

To further determine whether GSDME is functionally required for NDRV-induced pyroptosis, DEFs were transfected with GSDME-targeting siRNA prior to NDRV infection (S2A and S2B Fig). GSDME knockdown markedly reduced the generation of the cleaved GSDME-NT fragment at both 24 hpi and 48 hpi, accompanied by significantly decreased IL-1β secretion and LDH release compared with the siNC + NDRV group (Fig 2H–J). These findings provide direct loss-of-function evidence supporting a critical role for GSDME in mediating NDRV-induced pyroptotic responses.

### NDRV induces mtDNA oxidation and cytosolic release to trigger NLRP3 inflammasome activation

**Fig 3:**
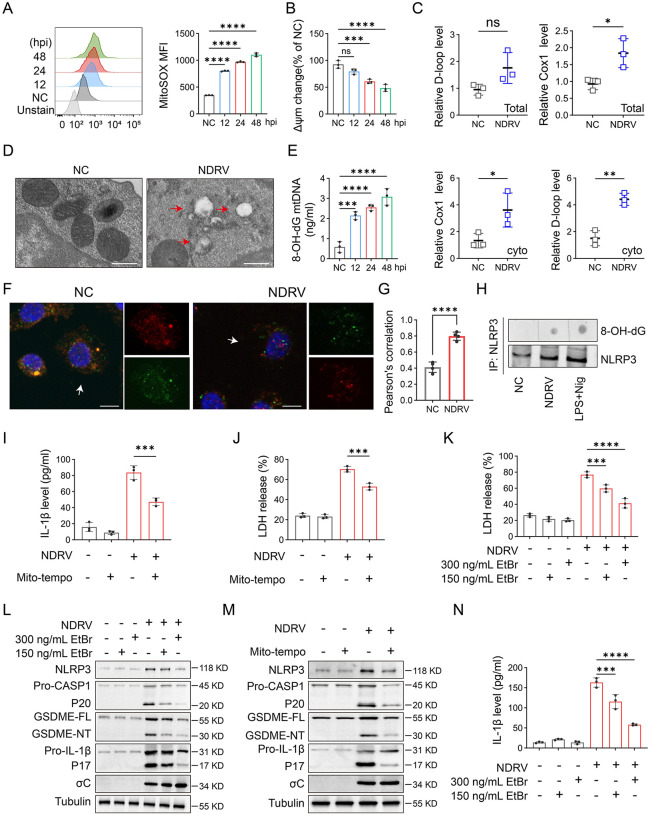
NDRV infection induces mitochondrial damage and oxidized mtDNA-mediated activation of the NLRP3 inflammasome. **(A)** Flow cytometry histograms of NDRV-infected PBMCs stained with MitoSOX Red (left) and quantification of the MFI in MitoSOX Red-stained NDRV-infected PBMCs (right). **(B)** ΔΨ_m_ changes in NDRV-infected PBMCs were assessed using the TMRM probe. **(C)** Total and cytosolic mtDNA levels in PBMCs following NDRV infection were quantified by qPCR and normalized to those in mock-infected controls. **(D)** Representative TEM images showing mitochondrial ultrastructural changes in PBMCs following NDRV infection. Red arrows indicate damaged mitochondria. Scale bar, 300 nm. **(E)** Cytosolic 8-OH-dG levels in the mtDNA fraction from NDRV-infected PBMCs were measured by ELISA. **(F)** Immunofluorescence staining of NLRP3 (green), 8-OH-dG (red), and DAPI (blue) in PBMCs at 24 hpi. Arrows indicate NLRP3 and 8-OH-dG colocalization. Scale bar, 5 μm. **(G)** Quantification of NLRP3 and 8-OH-dG colocalization by Pearson’s correlation coefficient. **(H)** PBMCs were mock-treated, infected with NDRV, or treated with LPS plus nigericin as indicated. Cell lysates were subjected to IP with an anti-NLRP3 antibody followed by dot blotting with an anti-8-OH-dG antibody. **(I)** IL-1β concentrations in culture supernatants from NDRV-infected PBMCs with or without Mito-TEMPO treatment. **(J)** LDH release from NDRV-infected PBMCs with or without Mito-TEMPO treatment. **(K)** LDH release from NDRV-infected PBMCs with or without EtBr treatment. **(L)** WB analysis of NLRP3, CASP-1, GSDME, IL-1β, and σC in NDRV-infected PBMCs with or without EtBr treatment. Tubulin was used as the loading control. **(M)** WB analysis of NLRP3, CASP-1, GSDME, IL-1β, and σC in NDRV-infected PBMCs with or without Mito-TEMPO treatment. Tubulin was used as the loading control. **(N)** IL-1β concentrations in culture supernatants from NDRV-infected PBMCs with or without EtBr treatment. Data are shown as mean ± SD. Statistical significance was determined by an unpaired Student’s t-test for two-group comparisons or ANOVA followed by Tukey’s multiple-group comparisons. ns, not significant; *p < 0.05, **p < 0.01, ***p < 0.001, ****p < 0.0001.

To identify upstream signals responsible for NLRP3 inflammasome activation, we examined mitochondrial integrity in infected PBMCs. Flow cytometry revealed a pronounced increase in MitoSOX fluorescence, indicating substantial accumulation of mtROS (Fig 3A). Conversely, TMRM fluorescence intensity was markedly reduced, indicating a loss of mitochondrial membrane potential (ΔΨ_m_) (Fig 3B). TEM analysis further showed mitochondrial swelling and structural disruption in NDRV-infected PBMCs (Fig 3D). These results indicate that NDRV infection severely compromises mitochondrial function.

We next investigated whether this dysfunction leads to mtDNA oxidation and subsequent release into the cytosol. qPCR analysis showed an overall increase in total mtDNA abundance and a significant elevation of cytosolic mtDNA levels in infected PBMCs (Fig 3C). Subsequent ELISA analysis detected increased 8-hydroxy-2’-deoxyguanosine (8-OH-dG), a marker of oxidative damage, in the cytosolic mtDNA fraction (Fig 3E), demonstrating that NDRV induces mtDNA oxidation and cytosolic translocation.

To determine the association between ox-mtDNA and NLRP3, cell lysates were subjected to immunoprecipitation (IP) using an anti-NLRP3 antibody, followed by dot blotting for 8-OH-dG. This analysis confirmed the presence of ox-mtDNA species within the NLRP3 precipitates (Fig 3H, S3A and S3B Fig). Immunofluorescence (IF) staining further demonstrated clear cytosolic colocalization of ox-mtDNA with NLRP3 (Fig 3F), and quantitative colocalization analysis confirmed increased NLRP3–8-OH-dG colocalization following NDRV infection (Fig 3G). Functionally, scavenging mtROS with Mito-TEMPO significantly reduced IL-1β secretion, LDH release, and activation of the CASP-1/GSDME axis (Fig 3I, 3J and 3M). Similarly, depleting mtDNA using ethidium bromide (EtBr) attenuated NDRV-induced LDH release, IL-1β secretion, and CASP-1/GSDME activation (Fig 3K, 3L, 3N, S3C, and S3D Fig).

Collectively, these results demonstrate that NDRV infection induces mitochondrial dysfunction, leading to the release of ox-mtDNA, which subsequently activates NLRP3 to drive inflammasome activation.

### The viral σC protein is sufficient to trigger mitochondrial stress and pyroptosis

To confirm that DEFs are also susceptible to NDRV-induced pyroptosis, DEFs were infected with NDRV and examined for pyroptosis-associated changes. Similar to the observations in PBMCs, NDRV infection in DEFs induced increased LDH release, elevated IL-1β secretion, enhanced PI uptake, and activation of the CASP-1/GSDME signaling pathway (S4A–E Fig). These findings demonstrate that NDRV infection triggers pyroptotic responses in DEFs and further validate the use of DEFs for subsequent σC functional analyses.

**Fig 4:**
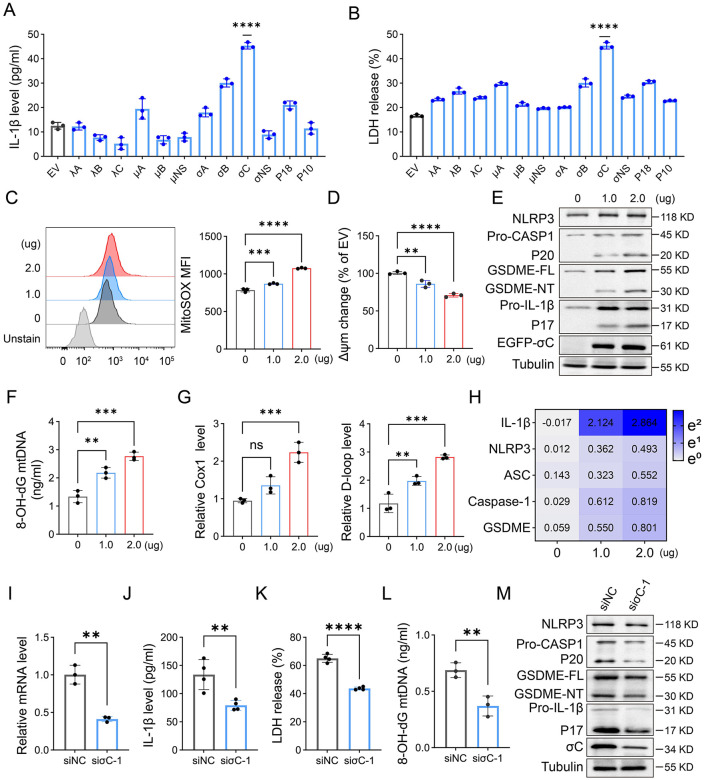
σC is a key viral factor driving NDRV-induced mitochondrial damage and pyroptosis. **(A–B)** DEFs were transfected with pEGFP-C3 expression plasmids encoding individual NDRV proteins. Protein expression of the screened constructs was verified by WB analysis (S5A Fig). IL-1β concentrations in the supernatants were quantified by ELISA (A), and LDH release was measured (B). **(C–H)** DEFs were transfected with pEGFP-C3-σC or empty vector for 48 h. **(C)** Representative flow cytometry histograms of MitoSOX Red staining (left) and corresponding MFI quantification (right). **(D)** ΔΨ_m_ changes were assessed using the TMRM probe. **(E)** WB analysis of NLRP3, CASP-1, GSDME, IL-1β, and EGFP-σC expression. Tubulin was used as the loading control. **(F)** Cytosolic 8-OH-dG levels were quantified by ELISA. **(G)** Cytosolic mtDNA levels were measured by qPCR using Cox1 and D-loop primers and normalized to those in empty vector control cells. **(H)** Heatmap showing the relative mRNA expression profiles of the indicated inflammatory and pyroptosis-related genes in DEFs. Values are presented on a logarithmic scale, with deeper blue indicating higher expression than that in empty vector control cells. **(I–M)** DEFs were transfected with σC-targeting siRNA (siσC-1), with siNC as the negative control. At 8 h post-transfection, cells were infected with NDRV (MOI = 1.0). **(I)** σC mRNA levels were quantified by RT-qPCR at 24 hpi to validate knockdown efficiency. **(J)** IL-1β concentrations in the supernatants were quantified by ELISA. **(K)** LDH release was measured. **(L)** Levels of 8-OH-dG in cytosolic mtDNA were quantified by ELISA. **(M)** WB analysis of NLRP3, CASP-1, GSDME, IL-1β, and σC expression. Tubulin was used as the loading control. The data are presented as the mean ± SD values. Statistical significance was determined by ANOVA followed by Tukey’s post-hoc test for multiple-group comparisons and unpaired Student’s t-test for two-group comparisons. ns, not significant; *p < 0.05, **p < 0.01, ***p < 0.001, ****p < 0.0001.

To identify the specific viral determinants of pyroptosis, twelve structural and nonstructural proteins of NDRV were individually overexpressed in duck embryo fibroblasts (DEFs), and their expression was verified by WB analysis (S5A Fig). Overexpression of the σC protein elicited the highest levels of LDH release (Fig 4B) and IL-1β secretion (Fig 4A), implicating σC as a dominant driver of cytotoxic inflammatory responses. Transcriptional profiling revealed that σC expression alone upregulated inflammatory and pyroptosis-related genes (Fig 4H). WB analysis detected increased NLRP3 expression, enhanced CASP-1 processing, GSDME cleavage, and IL-1β maturation in σC-expressing cells (Fig 4E). Considering that mitochondrial injury is an upstream driver of inflammasome activation, we examined mitochondrial integrity in σC-expressing cells. Flow cytometry showed that σC expression increased mtROS levels (Fig 4C), reduced ΔΨ_m_ (Fig 4D), and promoted cytosolic accumulation of oxidized mtDNA and total mtDNA, as indicated by increased cytosolic 8-OH-dG, Cox1, and D-loop signals (Fig 4F and 4G). Collectively, these findings identify the viral σC protein as a key factor that is sufficient to trigger mitochondrial dysfunction and the resulting ox-mtDNA-mediated danger signaling, positioning it upstream of NLRP3 inflammasome activation.

### σC promotes NDRV-induced mitochondrial injury and pyroptosis during infection

To further assess the role of σC during infection, DEFs were transfected with σC-targeting siRNA (siσC-1) or a negative control siRNA (siNC) and subsequently infected with NDRV. RT-qPCR confirmed that 100 nM siσC-1 significantly reduced σC mRNA levels at 24 hpi (Fig 4I), with knockdown efficiency further validated at 48 h post-transfection (S5B and S5C Fig). In NDRV-infected cells, σC knockdown reduced mtROS accumulation and partially restored ΔΨm (S5D and S5E Fig), accompanied by decreased 8-OH-dG levels in cytosolic mtDNA (Fig 4L), IL-1β secretion (Fig 4J), and LDH release (Fig 4K). Immunoblotting further showed that σC knockdown dampened NLRP3 expression and reduced CASP-1 and IL-1β processing, together with decreased GSDME activation, while σC protein levels were reduced as expected (Fig 4M). Collectively, these results support that σC promotes mitochondrial damage–associated danger signaling and downstream pyroptosis during NDRV infection.

### σC interacts with COQ6 and disrupts its mitochondrial localization

**Fig 5:**
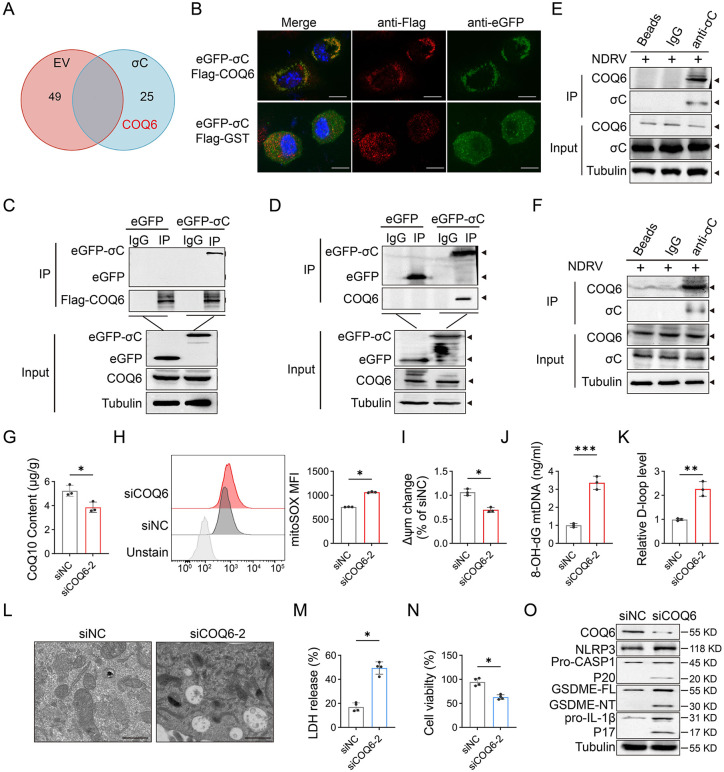
σC interacts with the host protein COQ6 to drive mitochondrial damage and pyroptosis. **(A)** Venn diagram comparing proteins identified by IP–MS in DEFs transfected with pEGFP-C3-σC or empty vector. **(B–C)** HEK-293T cells were co-transfected with pEGFP-σC and Flag-COQ6 or empty vector. **(B)** Confocal microscopy image showing the colocalization of Flag-COQ6 (red) and EGFP-σC (green), with Flag-GST included as an unrelated protein control. Scale bar, 5 μm. **(C)** Whole-cell lysates (WCLs) from 293T cells were subjected to IP with anti-Flag beads and immunoblotting with an anti-EGFP antibody. **(D)** WCLs from DEFs transfected with pEGFP-σC or empty vector were subjected to IP with anti-EGFP beads and immunoblotting with an anti-COQ6 antibody. **(E–F)** DEFs (E) or PBMCs (F) were infected with NDRV (MOI = 2) for 36 h. Lysates were subjected to IP using an anti-COQ6 antibody, rabbit IgG, or beads alone prior to immunoblotting. **(G–O)** DEFs were transfected with COQ6-targeting siRNAs or with siNC as the negative control. **(G)** Intracellular CoQ10 levels were quantified by targeted mass spectrometry. **(H)** Representative flow cytometry histograms of MitoSOX Red staining (left) and corresponding MFI quantification (right). **(I)** ΔΨ_m_ was assessed using the TMRM probe. **(J)** The amount of cytosolic 8-OH-dG was measured by ELISA. **(K)** Cytosolic DNA was extracted, and mtDNA levels were quantified by qPCR and normalized to those in the siNC control-transfected cells. **(L)** TEM images showing mitochondrial morphology. Scale bar, 500 nm. **(M)** LDH release was measured. **(N)** Cell viability was evaluated using a CCK-8 assay. **(O)** WB analysis of COQ6, NLRP3, CASP-1, GSDME, and IL-1β in DEFs transfected with siNC or COQ6-targeting siRNA. Tubulin was used as the loading control. The data are presented as the mean ± SD values. Statistical significance was determined by unpaired Student’s t-test for two-group comparisons or ANOVA followed by Tukey’s post-hoc test for multiple-group comparisons. *p < 0.05, **p < 0.01, ***p < 0.001, ****p < 0.0001.

To elucidate the precise molecular mechanism by which the viral σC protein induces mitochondrial injury and consequent pyroptosis, we performed immunoprecipitation-mass spectrometry (IP–MS) in DEFs transfected with pEGFP-C3-σC or empty vector to systematically identify interacting host proteins (Fig 5A and S6A Fig). The host protein COQ6 was prioritized for subsequent validation (S6B Fig). We first validated the physical interaction between σC and COQ6 by coexpressing Flag-tagged duck COQ6 and EGFP-tagged σC in 293T cells. Confocal microscopy confirmed the colocalization of σC and COQ6 within cells, whereas the Flag-tagged unrelated protein control GST did not show obvious colocalization with COQ6 (Fig 5B). Anti-Flag co-IP successfully recovered EGFP-σC (Fig 5C). In DEFs transfected with pEGFP-σC, anti-EGFP IP recovered endogenous COQ6 (Fig 5D). Crucially, the interaction was confirmed under infection conditions: IP using a monoclonal anti-COQ6 antibody also recovered the σC protein in both NDRV-infected DEFs and PBMCs (Fig 5E and 5F). In addition, GST pull-down assays further demonstrated a direct interaction between σC and COQ6 in vitro (S6C Fig), further supporting the specificity of the σC–COQ6 association.

We next examined the cellular consequences of COQ6 deficiency in DEFs (S6D, S6E and S6F Fig). siRNA-mediated knockdown of COQ6 reduced intracellular CoQ10 levels (Fig 5G), resulting in increased mtROS (Fig 5H), reduced ΔΨ_m_ (Fig 5I), elevated cytosolic 8-OH-dG (Fig 5J), and increased cytosolic mtDNA accumulation (Fig 5K and S6G Fig). Furthermore, TEM revealed significant mitochondrial ultrastructural damage following COQ6 knockdown (Fig 5L). Functionally, COQ6 deficiency reduced cell viability and enhanced pyroptosis-associated signaling (Fig 5M–O and S6H Fig). These data confirm that COQ6 is vital for maintaining mitochondrial homeostasis and preventing pyroptosis.

**Fig 6:**
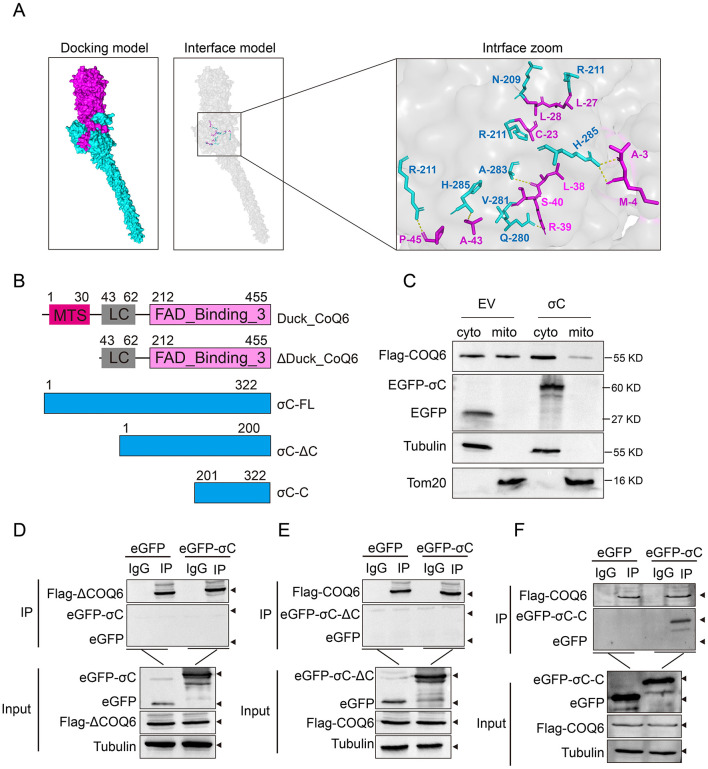
σC disrupts mitochondrial targeting of COQ6 by binding to its mitochondrial targeting signal. **(A)** AlphaFold3-based predictive modeling of the COQ6-σC interaction. Blue‐labeled residues correspond to σC, whereas the magenta‐labeled residues correspond to COQ6. **(B)** Schematic representation of σC and COQ6 truncation constructs. **(C)** DEFs were co-transfected with pEGFP-σC and Flag-COQ6, followed by cytosolic and mitochondrial fractionation. COQ6 protein levels in the cytosolic and mitochondrial fractions were measured by WB. TOM20 and Tubulin were used as mitochondrial and cytosolic markers, respectively. **(D)** HEK-293T cells were co-transfected with pEGFP-σC and Flag-ΔCOQ6 or empty vector, and whole-cell lysates from the 293T cells were immunoprecipitated with anti-Flag beads and immunoblotted with anti-EGFP. **(E)** HEK-293T cells were co-transfected with pEGFP-σC-ΔC and Flag-COQ6 or empty vector, and whole-cell lysates from 293T cells were immunoprecipitated with anti-Flag beads and immunoblotted with anti-EGFP. **(F)** HEK-293T cells were co-transfected with pEGFP-σC-C and Flag-COQ6 or empty vector, and whole-cell lysates from 293T cells were subjected to IP with anti-Flag beads and immunoblotting with an anti-EGFP antibody. Data are presented as the mean ± SD values. *p < 0.05, **p < 0.01, ***p < 0.001, ****p < 0.0001.

To map the interaction interface, AlphaFold3-based structural modeling predicted that the C-terminus of σC directly interacts with the mitochondrial targeting sequence (MTS) of COQ6 (Fig 6A). Guided by this prediction, we generated a series of truncation mutants for both σC and COQ6 (Fig 6B). Co-IP analysis showed that deleting the COQ6 MTS (Flag-ΔCOQ6) abolished binding to σC (Fig 6D). Conversely, the C-terminus of σC was both necessary and sufficient for the interaction (Fig 6E and 6F). These results identify the COQ6 MTS and the σC C-terminus as key determinants of the σC–COQ6 interaction. Subcellular fractionation demonstrated that σC expression significantly reduced COQ6 abundance in the mitochondrial fraction (Fig 6C), indicating that σC targets the COQ6 MTS to disrupt its mitochondrial localization.

### CoQ10 supplementation restores mitochondrial homeostasis and suppresses pyroptosis

**Fig 7:**
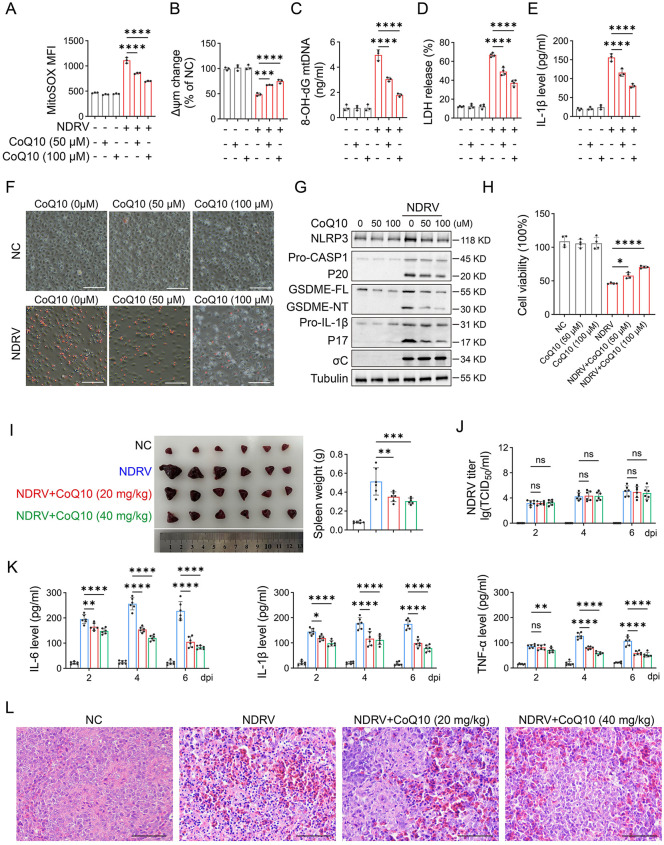
CoQ10 treatment alleviated NDRV-induced pyroptosis by restoring mitochondrial function and reducing the release of ox-mtDNA *in vitro* and *in vivo.* **(A–H)** PBMCs were mock-treated or infected with NDRV and were then treated with CoQ10 (50 or 100 μM). **(A)** Quantification of MitoSOX Red MFI in PBMCs. **(B)** ΔΨ_m_ changes in PBMCs assessed using the TMRM probe. **(C)** Cytosolic 8-OH-dG measured by ELISA. **(D)** LDH release was measured. **(E)** IL-1β concentrations in culture supernatants measured by ELISA. **(F)** Morphology images and PI uptake images of PBMCs. Scale bar, 50 μm. **(G)** WB analysis of NLRP3, CASP-1, GSDME, IL-1β, and σC in PBMCs. Tubulin was used as the loading control. **(H)** PBMC viability measured by CCK-8 assay. **(I–J)** Three-day-old ducklings were mock-infected or infected with NDRV and were then administered 20 mg/kg or 40 mg/kg CoQ10 by oral gavage daily. **(I)** Representative spleen images from ducklings in different treatment groups at 4 dpi and quantitative analysis of the spleen weight. **(J)** Infectious viral titers in spleen homogenates from different treatment groups at the indicated dpi were determined by TCID_50_ assay. **(K)** Serum concentrations of IL-6, IL-1β, and TNF-α in ducklings from different treatment groups were quantified by ELISA. **(L)** Representative H&E-stained images of duck spleens. Scale bar, 200 μm. Data are presented as mean ± SD. Statistical significance was determined by ANOVA followed by Tukey’s post-hoc test for multiple-group comparisons and unpaired Student’s t-test for two-group comparisons. ns, not significant; *p < 0.05, **p < 0.01, ***p < 0.001, ****p < 0.0001.

Since σC disrupts the COQ6–CoQ10 axis, we evaluated whether pharmacological supplementation with CoQ10 could mitigate NDRV-induced mitochondrial damage and pyroptosis-related responses in PBMCs. CoQ10 treatment significantly reduced mtROS accumulation (Fig 7A and S7A Fig), partially restored ΔΨm (Fig 7B), and decreased cytosolic 8-OH-dG levels (Fig 7C). Functionally, CoQ10 attenuated membrane damage, reducing LDH release (Fig 7D), PI uptake (Fig 7F), and IL-1β secretion (Fig 7E). Immunoblotting further showed that CoQ10 treatment dampened the NLRP3–CASP-1–GSDME cascade (Fig 7G), thereby preserving cell viability as measured by the CCK-8 assay (Fig 7H). These data collectively demonstrate that CoQ10 supplementation effectively limits virus-induced mitochondrial stress and the downstream pyroptotic responses *in vitro*.

### CoQ10 mitigates inflammatory responses without altering splenic viral loads

We finally evaluated the therapeutic potential of CoQ10 in NDRV-infected ducklings. Serum and spleen samples were collected at 2, 4, and 6 days post-infection (dpi) to assess inflammatory cytokines, splenic viral burden, and histopathology. CoQ10 treatment reduced splenic swelling and spleen weight at 4 dpi (Fig 7I), lowered serum levels of IL-6, IL-1β, and TNF-α (Fig 7K), and markedly alleviated splenic histopathology (Fig 7L). Notably, CoQ10 administration did not significantly alter either splenic infectious viral titers or viral RNA loads across the examined time points, as determined by TCID_50_ and RT-qPCR assays, respectively (Fig 7J and S7B Fig). These findings suggest that the protective effects of CoQ10 are primarily associated with the alleviation of host inflammatory injury under the current experimental conditions, rather than with a clear reduction in splenic viral burden.

## Discussion

The metabolic reprogramming of host cells is increasingly recognized as a central feature of viral pathogenesis [1,2]. Although pathogen sensing via pattern recognition receptors (PRRs) is well established, the specific mechanisms by which viruses repurpose host metabolic enzymes to drive immunopathology remain incompletely understood. In this study, using NDRV as a model of highly virulent viral infection, we define a pathogenic axis linking viral manipulation of bioenergetics to inflammatory cell death. We show that the outer-capsid protein σC functions beyond its canonical structural role, acting as a metabolic virulence factor that targets the host monooxygenase COQ6, an essential enzyme involved in CoQ10 biosynthesis [28–30]. This perturbation disrupts CoQ10-dependent respiration, induces mitochondrial dysfunction, and triggers the cytosolic accumulation of ox-mtDNA, which subsequently activates the NLRP3–CASP-1–GSDME signaling cascade. Consistent with this model, GSDME knockdown markedly attenuated NDRV-induced GSDME cleavage, IL-1β secretion, and LDH release, providing direct loss-of-function evidence for the functional involvement of GSDME in this pathway. Notably, σC knockdown during NDRV infection reduced cytosolic oxidized mtDNA and dampened NLRP3–CASP-1–GSDME activation, with decreased IL-1β secretion and LDH release. These findings support an essential role for σC in promoting mitochondrial injury-associated pyroptosis in infected cells. These findings provide a mechanistic basis for NDRV-associated injury and identify mitochondrial metabolic homeostasis as a tractable checkpoint for host-directed intervention.

A central advance of this work is the characterization of σC as a protein with metabolic regulatory functions. Classically, reovirus outer-capsid proteins are defined by their roles in attachment and serotype determination [26,27]. Our data expand this view, revealing that σC physically interacts with the N-terminal MTS of COQ6 to block its mitochondrial import. In addition to co-immunoprecipitation under overexpression and infection conditions, GST pull-down assays further supported a direct interaction between σC and COQ6, strengthening the specificity of this interaction. This interference creates a bottleneck in CoQ10 biosynthesis, driving the loss of ΔΨ_m_ and mtROS accumulation. These observations suggest that NDRV reshapes host bioenergetics by targeting a limiting step in mitochondrial metabolism rather than by encoding dedicated metabolic functions. Broadly, this aligns with the emerging concept that mitochondria represent a recurring vulnerability during viral infection [1,5], though the specific entry points vary. For instance, the mammalian orthoreovirus protein μ1 induces apoptosis via cytochrome c release [31], while SARS-CoV-2 ORF9b antagonizes TOM70 to dampen antiviral signaling [32]. In our avian model, σC acts specifically at the level of CoQ10 biosynthesis to promote ox-mtDNA release. By limiting mitochondrial COQ6 localization, σC promotes mtROS accumulation and ox-mtDNA release upstream of NLRP3 activation, consistent with the broader concept that mitochondrial DNA stress can shape innate immune responses, and that oxidized DNA fragments can exit mitochondria via mPTP- and VDAC-dependent routes to activate NLRP3 signaling [33–36]. Thus, different viruses may converge on mitochondrial dysfunction and inflammatory outcomes through mechanistically distinct “handles” on mitochondrial homeostasis.

The targeting of COQ6 by σC reveals a specific strategy for disrupting the CoQ10 pathway and mitochondrial-immune balance. As a pivotal monooxygenase, COQ6 is indispensable for the production of CoQ10, a lipid that not only functions as an electron carrier but also plays a critical role in regulating the mitochondrial permeability transition pore (mPTP) [37–39]. Our findings indicate that impairment of COQ6 by σC mimics the metabolic consequences associated with primary CoQ10 deficiency disorders, leading to the release of ox-mtDNA into the cytosol. This metabolic disturbance directly promotes NLRP3 inflammasome assembly. The disruption of COQ6 highlights the fragility of this metabolic pathway during infection.

The avian host provides a unique evolutionary context for dissecting GSDME-dependent pathology. In mammals, multiple pore-forming GSDMs can complicate the interpretation of inflammasome-driven membrane injury [40–44]. Ducks naturally lack the GSDMD pathway and primarily express GSDMA and GSDME [21,22]. This simplified system helps clarify how mitochondrial stress translates into NLRP3-dependent GSDME cleavage and inflammatory cell death, offering insights that may be applicable to mammalian contexts in which GSDME is highly expressed or preferentially engaged under specific pathological conditions. Recent studies have suggested that avian and lower vertebrate gasdermin utilization may differ from the canonical mammalian GSDMD-centered pathway. Billman et al. reported that avian GSDMA can be cleaved by caspase-1 in a heterologous overexpression system, supporting a potential CASP-1–GSDMA axis in birds [45]. In contrast, our study investigated endogenous pyroptotic signaling in native avian cells during NDRV infection. Under our experimental conditions, we did not detect obvious GSDMA cleavage within 48 hpi, whereas GSDME knockdown significantly reduced pyroptosis-associated responses. In addition, previous studies in lower vertebrate and avian systems have shown that GSDME can also function as a caspase-dependent pyroptotic executor under certain conditions [46,47]. These observations suggest that gasdermin usage in non-mammalian species may be context-dependent and influenced by cell type, stimulus, infection stage, and pathogen-specific signaling. Therefore, while our data support GSDME as a major terminal effector in the NDRV infection model, the potential involvement of GSDMA under other conditions cannot be fully excluded.

From a therapeutic perspective, our findings support the concept of disease tolerance, a defense strategy fundamentally distinct from antiviral resistance [48]. Resistance aims to eliminate the pathogen, whereas tolerance mechanisms minimize tissue damage to preserve host fitness. In our model, CoQ10 supplementation restored mitochondrial homeostasis and attenuated splenic pathology without significantly reducing viral RNA loads or infectious viral titers in tissues. This functional uncoupling of viral burden from tissue injury suggests that disease severity may be influenced not only by viral replication but also by the host’s capacity to tolerate metabolic and inflammatory stress. Recent frameworks propose that metabolism serves as a central regulator of tissue tolerance [49]. By replenishing the metabolic deficit created by σC, CoQ10 stabilizes the mitochondrial redox checkpoint. This suggests that bolstering metabolic resilience may represent a viable host-directed strategy, particularly in infections where damage is driven by dysregulated host responses rather than uncontrolled viral proliferation.

Several questions remain to be addressed. Given the cellular heterogeneity of PBMCs, future work will be required to define the specific immune subsets that drive NLRP3 activation and GSDME-dependent membrane injury. In addition, although ox-mtDNA is a prominent signal in our model, other mitochondrial danger cues may contribute in parallel and warrant further investigation. Finally, generating σC mutants using reverse genetics that are selectively defective in COQ6 binding while retaining viral infectivity would strengthen the causal evidence for the proposed mechanism. However, since σC is central to receptor recognition and entry, obtaining such entry-competent separation-of-function mutants remains technically challenging.

**Fig 8:**
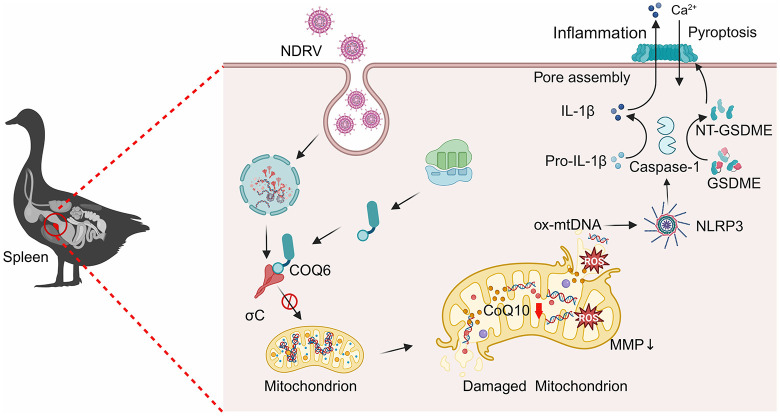
Proposed model for how the NDRV σC protein targets COQ6 to drive ox-mtDNA–NLRP3–CASP-1-GSDME–dependent pyroptosis. Created in BioRender. Yan, **H.** (2026) https://BioRender.com/x9t99i0.

In summary, this study defines a σC–COQ6–CoQ10–ox-mtDNA–NLRP3 axis that links viral metabolic reprogramming to inflammasome-driven inflammatory cell death. We reveal how a structural protein of an avian orthoreovirus targets a conserved metabolic enzyme to disrupt mitochondrial homeostasis, thereby promoting inflammatory tissue injury. Beyond clarifying NDRV pathogenesis, these findings illustrate a broader principle by which avian orthoreoviruses may exploit mitochondrial vulnerabilities to shape disease progression (Fig 8).

## Materials and methods

### Ethics statement

Animal experiments were conducted in strict accordance with the institutional guidelines and were approved by Shandong Agricultural University Animal Care and Use Committee (Approval Number: SDAUA-2025–263). All personnel involved in animal handling and euthanasia were appropriately trained.

### Animal welfare

No surgical procedures were performed in this study. Animal health and behavior were monitored at least twice daily throughout the experiments. Humane endpoints were predefined, including severe lethargy or unresponsiveness, inability to stand or access food/water, severe respiratory distress, severe dehydration, rapid body weight loss, or any other signs of severe distress. Animals reaching humane endpoints would have been euthanized immediately. No animals reached these predefined humane endpoints before scheduled euthanasia, and no animals were found dead during the study. At scheduled endpoints, ducklings were euthanized by intraperitoneal injection of an overdose of pentobarbital sodium, followed by sample collection. All efforts were made to minimize animal suffering.

### Animal maintenance and viral challenge model

Twenty-four three-day-old specific pathogen-free (SPF) ducklings were obtained from Shandong Haotai Laboratory Animal Breeding Co., Ltd. and housed in isolators with *ad libitum* access to food and water. Ducklings were randomly assigned to two groups using a random number generator. Ducklings received an intramuscular injection of NDRV suspension (0.5 mL, 10⁵ TCID₅₀/mL) or sterile PBS as the control. At 24, 48, and 72 hours post-infection (hpi), four ducklings from each group were euthanized, and serum and spleen tissues were collected for downstream analyses.

### Cell culture and viral propagation

HEK293T and DF-1 cells (ATCC, Manassas, VA, USA) were maintained in Dulbecco’s modified Eagle Medium (DMEM; Gibco) supplemented with 10% fetal bovine serum (FBS; Biological Industries, Israel), 100 U/mL penicillin, and 100 μg/mL streptomycin at 37°C in a 5% CO_2_. Duck primary peripheral blood mononuclear cells (PBMCs) were isolated from 5-day-old SPF ducklings using Ficoll-Paque density gradient centrifugation (Cytiva, Sweden) and cultured in RPMI-1640 with 10% FBS. The NDRV N19 strain (GenBank: MZ558210.1) was propagated in DF-1 cells. Viral titers were calculated using the Reed-Muench method and expressed as the median tissue culture infective dose (TCID_50_). For *in vitro* infection, cells were inoculated with NDRV at the indicated multiplicity of infection (MOI) for 1 h, washed with PBS, and maintained in DMEM containing 2% FBS.

### Plasmid construction

Viral genes (λA, λB, λC, μA, μB, μNS, σA, σB, σC, σNS, P10, and P18) were amplified from N19 cDNA and cloned into the pEGFP-C3 vector. For functional assays, σC and duck COQ6 were cloned into the pCAGGS-Flag vector. Truncated mutants were generated by PCR using full-length plasmids as templates. All constructs were verified by Sanger sequencing. Primer sequences are listed in S1 Table.

### RNA interference (siRNA) and transfection

Transient knockdown of COQ6, σC and GSDM was performed using specific siRNAs (GenePharma, Shanghai, China). DEFs were transfected with siRNAs or a non-targeting control using Lipofectamine 3000 (Invitrogen) at ~50% confluence. At 24 h post-transfection, cells were infected with NDRV. Samples were collected at the indicated time points post-infection. Knockdown efficiency was assessed by RT-qPCR and WB. siRNA sequences are provided in S2 Table.

### Inhibition treatment

For inflammasome inhibition, PBMCs were pretreated with the NLRP3 inhibitor MCC950 (50 μM) or the CASP-1 inhibitor VX-765 (50 μM) for 30 min prior to NDRV infection (MOI = 5.0). After a 1-h adsorption period, the inoculum was removed, and cells were maintained in fresh RPMI-1640 containing 2% FBS for 36 h.

### *In vitro* CoQ10 rescue assay

To evaluate the protective efficacy of CoQ10 on mitochondrial function and cell viability, PBMCs were infected with NDRV (MOI = 5.0). Following a 1-h adsorption period at 37°C, the inoculum was removed, and cells were washed with PBS. Cells were subsequently maintained in RPMI-1640 supplemented with 2% FBS containing CoQ10 (MedChemExpress, NJ, USA; purity 99.69%) at final concentrations of 50 μM or 100 μM. A stock solution of CoQ10 was prepared in anhydrous ethanol, and an equivalent volume of vehicle (ethanol) was added to the mock-treated and NDRV-only control groups. Cells were harvested at 36 hpi to assess mtROS levels, mitochondrial membrane potential, and pyroptosis markers as described below.

### Immunoprecipitation and mass spectrometry (IP–MS)

DEFs transfected with pEGFP-σC or empty vector were washed with cold PBS and lysed in IP buffer containing protease and phosphatase inhibitors. After centrifugation, lysates were incubated with an anti-GFP antibody overnight at 4°C, followed by Protein A/G agarose beads for 2 h. Beads were washed four times with high-salt buffer. Eluted proteins were subjected to trypsin digestion and identified by LC–MS/MS (GeneCreate, Guangzhou, China). Detailed information for the antibodies is provided in S5 Table.

### Co-Immunoprecipitation (Co-IP) and dot blotting

Cell lysates were incubated with the indicated primary antibodies against Flag, Myc, or COQ6 overnight, followed by Protein A/G beads. For the detection of oxidized DNA in protein complexes, eluates were spotted onto nitrocellulose membranes (Dot Blot), UV-crosslinked, and probed with an anti-8-OH-dG antibody.

### UPLC–MS/MS quantification of CoQ10

Cellular lipids were extracted using a mixture of anhydrous ethanol (200 µL) and isopropanol (1 mL). Samples were ultrasonicated for 30 min and centrifuged. The supernatants were dried under vacuum, reconstituted in isopropanol/methanol (1:1, v/v), and filtered through 0.22 µm membranes. CoQ10 levels were quantified by Ultra-Performance Liquid Chromatography-Tandem Mass Spectrometry (UPLC–MS/MS) based on external standard curves. CoQ10 levels were quantified based on external standard curves. CoQ10 abundance was normalized to the wet weight of each sample.

### Cytosolic DNA extraction

To assess mtDNA release, a selective permeabilization protocol was used. Infected cells were harvested at 36 hpi and resuspended in buffer containing 150 mM NaCl, 50 mM HEPES (pH 7.4), and 25 μg/mL digitonin. The mixture was incubated for 10 min at 4°C to permeabilize the plasma membrane. Nuclei were pelleted by centrifugation at 980 × g for 3 min. The supernatant was further clarified at 17,000 × g for 10 min to pellet mitochondrial debris, yielding the cytosolic fraction. DNA was extracted using DNeasy Blood & Tissue Kit (Vazyme, Nanjing, China). To minimize mitochondrial and nuclear contamination, cytosolic fractions were subjected to multiple washing and clarification steps. The purity of the cytosolic fractions was verified by WB analysis using TOM20 and Histone H3 as mitochondrial and nuclear markers, respectively, both of which showed negligible signals in the cytosolic fraction.

### Quantification of mtDNA levels

mtDNA levels were quantified by SYBR Green qPCR. Total mtDNA was normalized to the nuclear *Tert* gene. For cytosolic mtDNA, 20 ng of linearized EGFP plasmid was added to each sample prior to extraction as a spike-in control for normalization. Primers are listed in S3 Table. Primers were specifically designed to amplify mitochondrial DNA fragments without interference from nuclear mitochondrial pseudogenes.

### mtDNA depletion

To deplete mtDNA, PBMCs were cultured in medium supplemented with ethidium bromide (EtBr; 450 ng/mL) for 24 or 48 h prior to infection. EtBr-treated but uninfected cells were included as controls to evaluate the basal effect of EtBr treatment. Depletion efficiency was confirmed by qPCR before subjecting cells to NDRV infection (MOI = 5.0).

### MitoSOX measurement

The mtROS levels were assessed using MitoSOX Red (Thermo Fisher Scientific). Infected cells were washed with PBS and incubated with 5 µM MitoSOX for 20 min at 37°C. Fluorescence was analyzed by flow cytometry (BD FACSCanto II) and processed using FlowJo software.

### Mitochondrial membrane potential assay

Cells were incubated with 200 nM Tetramethylrhodamine methyl ester (TMRM; Thermo Fisher Scientific) for 30 min at 37°C. Cells were washed with PBS, and fluorescence intensity was measured using a BioTek Synergy H1 microplate reader.

### Western blotting

Cells were lysed in RIPA buffer containing protease inhibitors (Beyotime, Shanghai, China). Lysates were clarified by centrifugation at 12,000 rpm for 10 min at 4°C. Proteins were separated on SDS-PAGE and transferred to nitrocellulose membranes (Millipore). Membranes were blocked with 5% nonfat milk for 1.5 h, incubated with primary antibodies overnight at 4°C, and detected using HRP-conjugated secondary antibodies (1:5000) for 45 min. Signals were developed using ECL reagents and captured with a Fusion FX system (Vilber, France).

### RT-qPCR

Total RNA was extracted using TRNzol Universal Reagent (TIANGEN) and reverse-transcribed using the FastKing RT Kit. qPCR was performed on a LightCycler 96 system (Roche) using SYBR Green with specific primers listed in S4 Table. For host gene expression, relative mRNA levels were calculated using the 2^-ΔΔCt method with β-actin as the internal control. For viral-load quantification, NDRV RNA levels were determined by absolute qPCR targeting the viral σC gene, using a plasmid standard curve. Viral RNA loads in spleen tissues were normalized to sample weight and expressed as copies per mg tissue.

### ELISA

For cell supernatants, media were collected and then clarified by centrifugation. Blood was allowed to clot at room temperature, and serum was separated by centrifugation and stored at -80°C until ELISA. Cytokine levels (IL-6, TNF-α, IL-1β) in cell culture supernatants and serum were quantified using commercial ELISA kits (MLBIO, Shanghai, China). Cytosolic 8-OH-dG levels were measured using a specific ELISA kit (Sangon, Shanghai, China) according to the manufacturer’s instructions.

### LDH release assay

Culture supernatants were collected, clarified, and analyzed using an LDH Detection Kit (Beyotime, Shanghai, China) following the manufacturer’s protocol. After incubation at 37°C for 20 min in the dark, stop solution was added and absorbance was measured at 450 nm.

### PI staining

Propidium Iodide (PI; 1.0 μg/mL) was added to the culture medium. After incubation for 20 min in the dark, cells were imaged using a Nikon ECLIPSE Ts2R microscope.

### Cytotoxicity assays

Cells in 96-well plates were treated with CCK-8 reagent (Beyotime, Shanghai, China) for 3 h at 24, 48, or 72 hpi. Absorbance was measured at 450 nm.

### Immunofluorescence staining

Cells on coverslips were fixed with 4% paraformaldehyde for 15 min, permeabilized with 0.5% Triton X-100, and blocked with 10% goat serum for 30 min. Samples were incubated with primary antibodies overnight at 4°C, followed by fluorescent secondary antibodies for 1 h in the dark. Nuclei were stained with DAPI. Images were acquired using an Andor Dragonfly confocal microscope and analyzed using Imaris software.

### Histopathology and immunohistochemistry (IHC)

Spleen tissues were fixed in 4% paraformaldehyde, embedded in paraffin, and sectioned at 4 μm. For H&E staining, sections were deparaffinized, rehydrated, stained with hematoxylin for 3–5 min, differentiated in 1% acid alcohol for 5–10 s, rinsed, and counterstained with eosin. After dehydration and mounting with neutral resin, sections were examined using a Nikon microscope. For IHC, sections underwent antigen retrieval and were blocked with 10% BSA. Primary antibodies against NLRP3, IL-1β, NDRV-σC, CASP-1, or GSDME were applied overnight at 4°C. Detection was performed using a polymer-conjugated secondary antibody and DAB substrate.

### *In vivo* CoQ10 therapeutic trial

Seventy-two 3-day-old ducklings were randomly and equally assigned to four groups: PBS control, NDRV infection, NDRV + CoQ10 (20 mg/kg), NDRV + CoQ10 (40 mg/kg). Ducklings in the control group received intramuscular PBS (0.5 mL). Ducklings in all three NDRV-challenged group received intramuscular virus (0.5 mL; 10⁵ TCID₅₀/mL). CoQ10 (MedChemExpress) was suspended in a 0.5% sodium carboxymethylcellulose (CMC-Na) and freshly prepared before each treatment. Treatments were administered via oral gavage once daily, initiating immediately after infection. At 2, 4, and 6 dpi, six ducklings from each group were euthanized as described above, and serum and spleen tissues were collected for cytokine measurement, viral load quantification, infectious viral titer assay, and histopathology. Investigators assessing outcomes were blinded to group allocation.

### Statistical analysis

Data are presented as mean ± Standard Deviation (SD). Statistical analyses were performed using GraphPad Prism 9.0. Differences between groups were evaluated using the unpaired Student’s t-test or Analysis of Variance (ANOVA) followed by Tukey’s post-hoc test. Statistical significance was defined as *p < 0.05, **p < 0.01, ***p < 0.001, and ****p < 0.0001.

## Supporting information

S1 Fig IHC detection of NDRV σC protein in spleen sections at 48 hpi (related to Fig 1). Representative IHC images and quantification of the σC-positive area in mock- and NDRV-infected ducklings are shown. Scale bar, 25 μm. Data are presented as mean ± SD. Statistical significance was determined by unpaired Student’s t-test. ****p < 0.0001.
(TIF)

S2 Fig GSDME knockdown validation and LDH release after inhibitor treatment (related to Fig 2). **(A)** RT-qPCR validation of GSDME knockdown efficiency in DEFs transfected with three independent GSDME-targeting siRNAs. **(B)** WB validation of GSDME protein levels in DEFs transfected with three independent GSDME-targeting siRNAs. Tubulin was used as the loading control. **(C)** LDH release from NDRV-infected PBMCs with or without MCC950 treatment. **(D)** LDH release from NDRV-infected PBMCs with or without VX-765 treatment. Data are presented as mean ± SD. Statistical significance was determined by ANOVA followed by Tukey’s post-hoc test. ****p < 0.0001.
(TIF)

S3 Fig Cytosolic mtDNA release after NDRV infection and EtBr-mediated mtDNA depletion (related to Fig 3). **(A–B)** PBMCs were mock-treated, infected with NDRV, or treated with LPS plus nigericin as indicated. Cytosolic mtDNA levels were measured by qPCR using D-loop (A) and Cox1 (B) primers and normalized to mock-treated cells. **(C–D)** Cytosolic mtDNA levels in NDRV-infected PBMCs with or without EtBr treatment were measured by qPCR using Cox1 (C) and D-loop (D) primers and normalized to mock-treated cells. Data are presented as mean ± SD. Statistical significance was determined by ANOVA followed by Tukey’s post-hoc test. ns, not significant; *p < 0.05, **p < 0.01, ***p < 0.001.
(TIF)

S4 Fig NDRV infection induces pyroptosis in DEFs (related to Fig 4). **(A)** IL-1β concentrations in culture supernatants from NDRV-infected DEFs were measured by ELISA. **(B)** LDH release from NDRV-infected DEFs. **(C)** Cell viability of DEFs after NDRV infection was measured by CCK-8 assay. **(D)** Representative bright-field and PI staining images of DEFs after NDRV infection. Scale bar, 30 μm. **(E)** WB analysis of GSDME, IL-1β, NLRP3, CASP-1, and σC in NDRV-infected DEFs at the indicated time points. Tubulin was used as the loading control. Data are presented as mean ± SD. Statistical significance was determined by ANOVA followed by Tukey’s post-hoc test. **p < 0.01, ***p < 0.001, ****p < 0.0001.
(TIF)

S5 Fig Validation of viral protein expression, σC knockdown validation and effects on mitochondrial ROS and ΔΨm (related to Fig 4). **(A)** WB validation of the expression of the 12 overexpressed NDRV proteins in DEFs. Tubulin was used as the loading control. **(B)** RT-qPCR validation of σC knockdown efficiency in DEFs transfected with three independent σC-targeting siRNAs. **(C)** WB validation of σC protein levels in DEFs transfected with three independent σC-targeting siRNAs. Tubulin was used as the loading control. **(D–E)** DEFs were transfected with siσC-1, with siNC as the negative control, and then infected with NDRV. MitoSOX Red MFI was quantified to assess mtROS accumulation (D), and ΔΨm changes were measured using the TMRM probe (E). Data are presented as mean ± SD. Statistical significance was determined by ANOVA followed by Tukey’s post-hoc test for multiple-group comparisons or unpaired Student’s t-test for two-group comparisons. ns, not significant; *p < 0.05, ***p < 0.001, **p < 0.0001.
(TIF)

S6 Fig σC–COQ6 interaction, and COQ6 knockdown (related to Fig 5). **(A)** Top candidate host proteins identified by IP–MS as potential σC-interacting factors. COQ6 is highlighted in red. **(B)** Schematic illustration of the role of COQ6 in the CoQ10 biosynthetic pathway and mitochondrial redox homeostasis. Panel B was created in BioRender. Yan, H. (2026) https://BioRender.com/q0yte9c. **(C)** Direct interaction between GST-σC and His-COQ6 was validated by GST pull-down assay, followed by WB detection with anti-His and anti-GST antibodies. **(D)** RT-qPCR validation of COQ6 knockdown efficiency in DEFs transfected with three independent COQ6-targeting siRNAs. **(E)** WB validation of COQ6 protein levels in DEFs transfected with three independent COQ6-targeting siRNAs. Tubulin was used as the loading control. **(F)** RT-qPCR validation of COQ6 mRNA levels in DEFs transfected with siCOQ6–2. **(G)** Cytosolic mtDNA levels in COQ6-knockdown DEFs were measured by qPCR using Cox1 primers. **(H)** IL-1β concentrations in culture supernatants from COQ6-knockdown DEFs were measured by ELISA. Data are presented as mean ± SD. Statistical significance was determined by ANOVA followed by Tukey’s post-hoc test for multiple-group comparisons or unpaired Student’s t-test for two-group comparisons. ns, not significant; **p < 0.01, ***p < 0.001, ***p < 0.0001.
(TIF)

S7 Fig MitoSOX Red staining and NDRV viral load quantification (related to Fig 7). **(A)** PBMCs were mock-treated or infected with NDRV and then treated with CoQ10 (50 or 100 μM). Representative flow cytometry histograms of MitoSOX Red staining. **(B)** Viral loads in spleen homogenates from different treatment groups at the indicated dpi were determined by qPCR. Data are presented as mean ± SD. Statistical significance was determined by ANOVA followed by Tukey’s post-hoc test. ns, not significant; *p < 0.05.
(TIF)

S1 Table Primers used for constructing eukaryotic expression plasmids. (DOCX)

S2 Table siRNA sequences targeting σC, duck COQ6, and GSDME. (DOCX)

S3 Table Primers used for mtDNA quantification. (DOCX)

S4 Table Primers used for RT-qPCR. (DOCX)

S5 Table Detailed information for the antibodies used in this study. (DOCX)

S1 Raw Gel Raw images for the figures. (DOCX)

S1 Data Source data and statistical analyses. (XLSX)
