# Supplementary figures and images for: Novel duck reovirus σC hijacks the mitochondrial COQ6–CoQ10 axis to drive NLRP3-dependent pyroptosis
Source: PLoS Pathog. 2026 Jul 7;22(7):e1014392. doi: 10.1371/journal.ppat.1014392 (PMC13367899; doi:10.1371/journal.ppat.1014392)

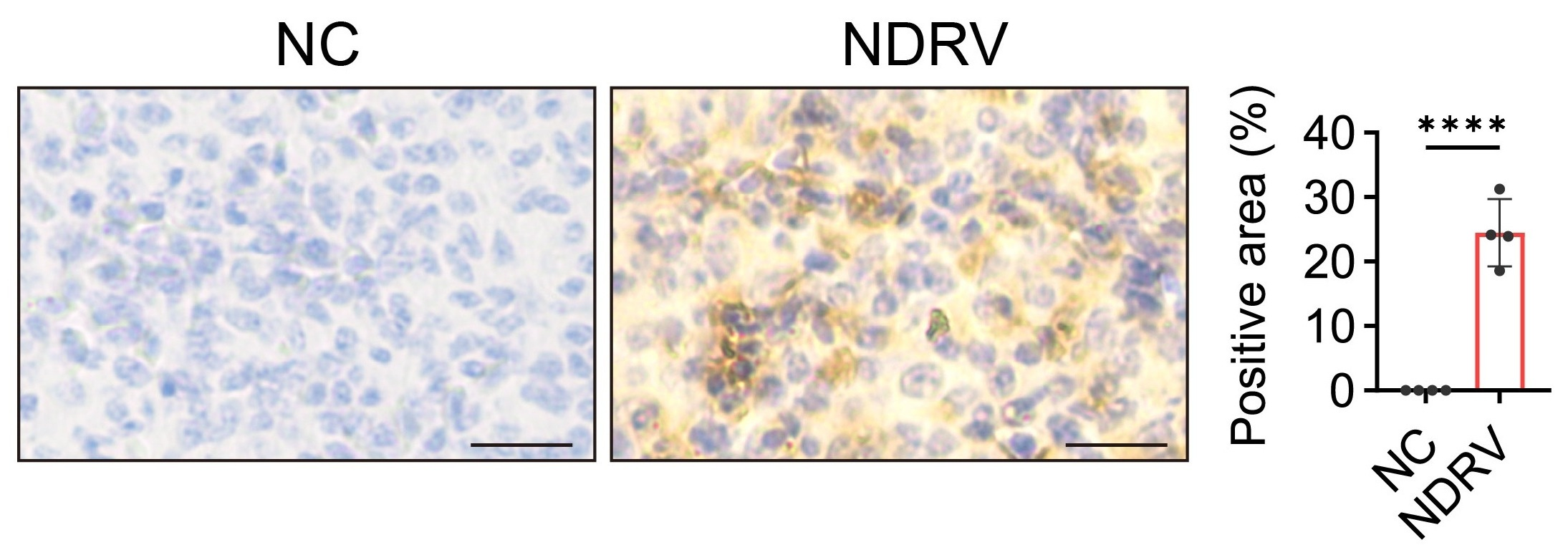

Supplement: S1 Fig — Representative IHC images and quantification of the σC-positive area in mock- and NDRV-infected ducklings are shown. Scale bar, 25 μm. Data are presented as mean ± SD. Statistical significance was determined by unpaired Student’s t-test. ****p < 0.0001. (TIF) [file ppat.1014392.s001.tif]

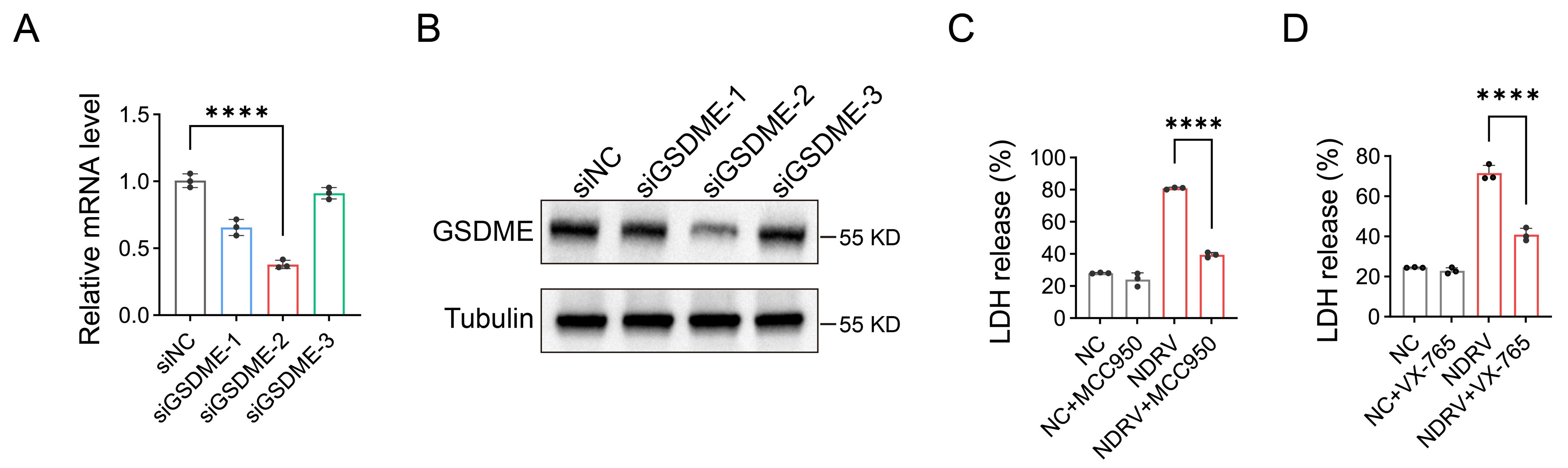

Supplement: S2 Fig — (A) RT-qPCR validation of GSDME knockdown efficiency in DEFs transfected with three independent GSDME-targeting siRNAs. (B) WB validation of GSDME protein levels in DEFs transfected with three independent GSDME-targeting siRNAs. Tubulin was used as the loading control. (C) LDH release from NDRV-infected PBMCs with or without MCC950 treatment. (D) LDH release from NDRV-infected PBMCs with or without VX-765 treatment. Data are presented as mean ± SD. Statistical significance was determined by ANOVA followed by Tukey’s post-hoc test. ****p < 0.0001. (TIF) [file ppat.1014392.s002.tif]

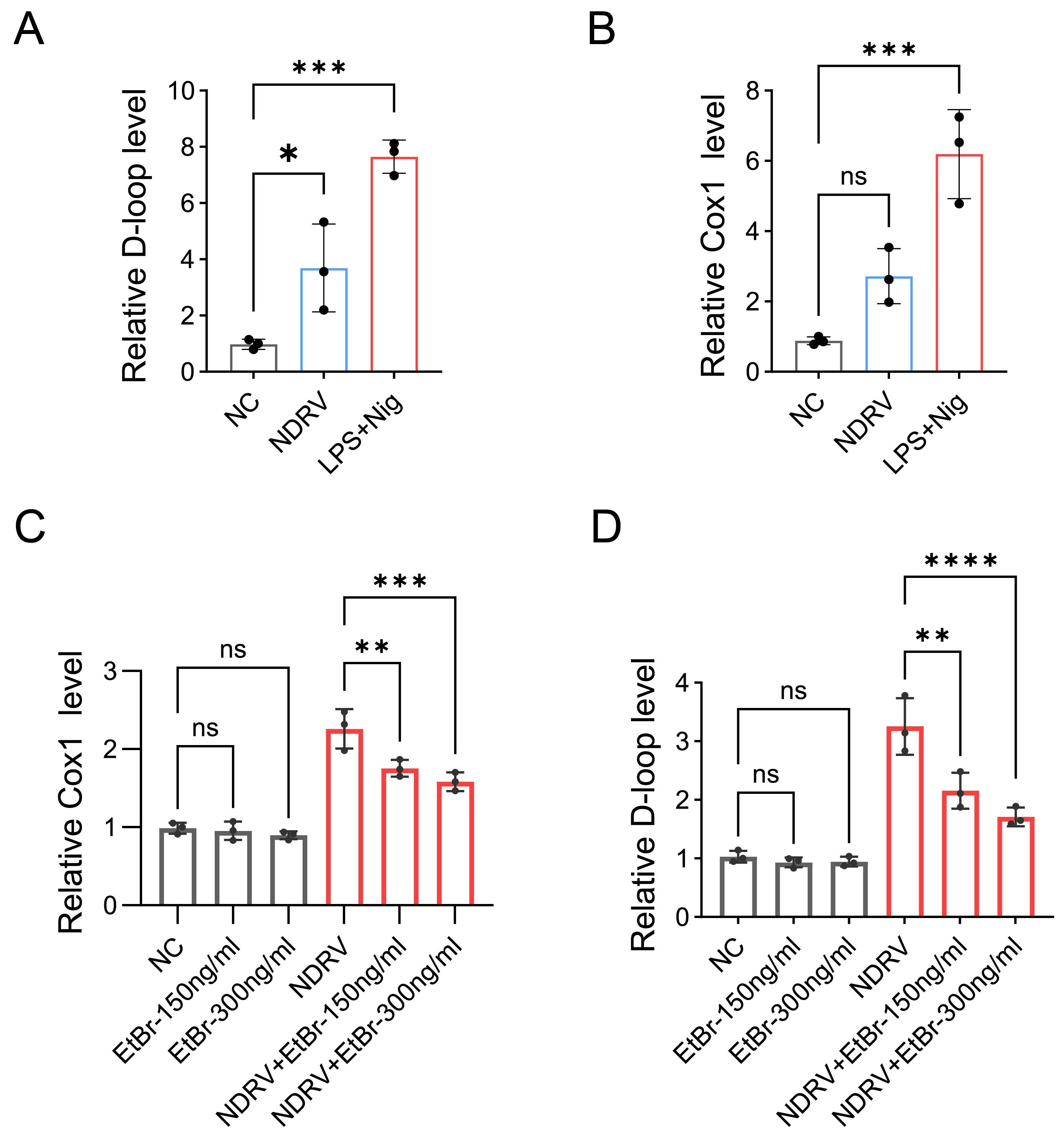

Supplement: S3 Fig — (A–B) PBMCs were mock-treated, infected with NDRV, or treated with LPS plus nigericin as indicated. Cytosolic mtDNA levels were measured by qPCR using D-loop (A) and Cox1 (B) primers and normalized to mock-treated cells. (C–D) Cytosolic mtDNA levels in NDRV-infected PBMCs with or without EtBr treatment were measured by qPCR using Cox1 (C) and D-loop (D) primers and normalized to mock-treated cells. Data are presented as mean ± SD. Statistical significance was determined by ANOVA followed by Tukey’s post-hoc test. ns, not significant; *p < 0.05, **p < 0.01, ***p < 0.001. (TIF) [file ppat.1014392.s003.tif]

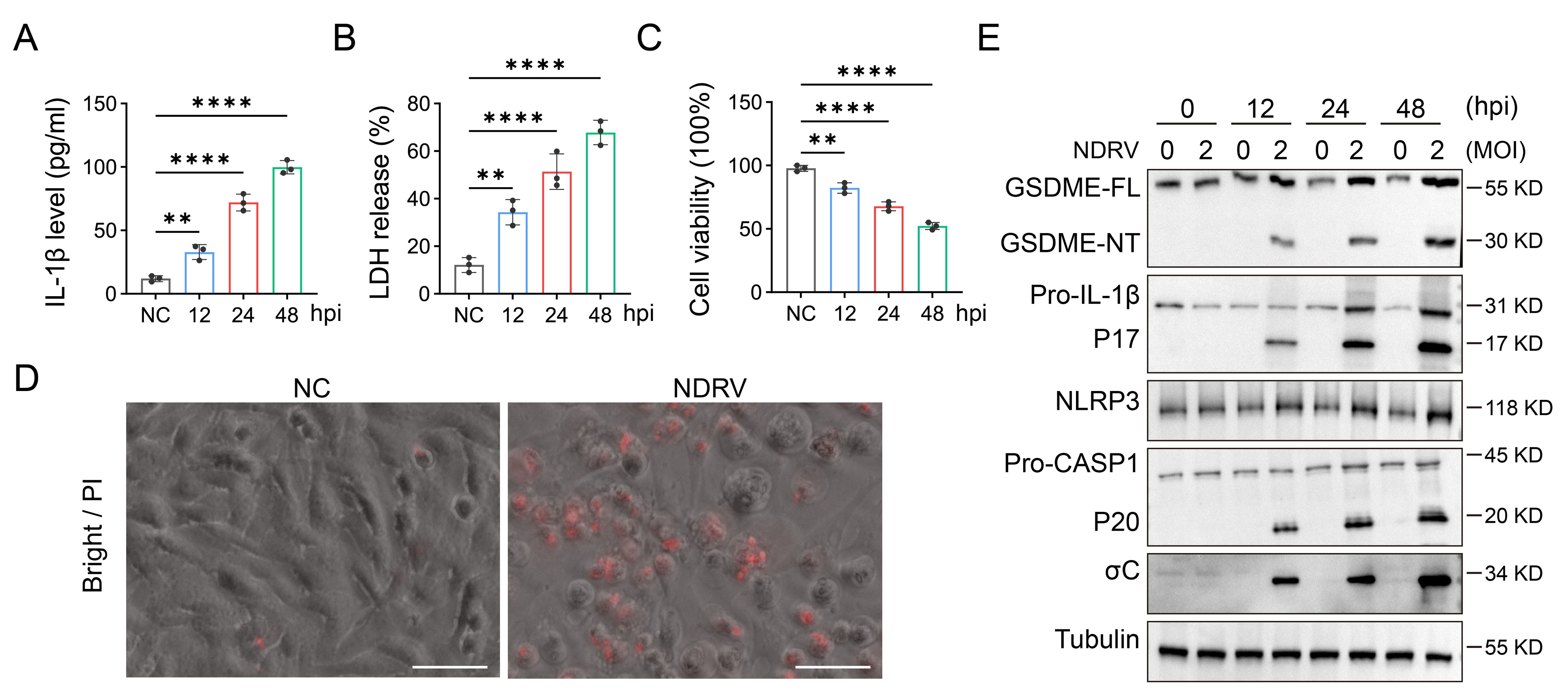

Supplement: S4 Fig — (A) IL-1β concentrations in culture supernatants from NDRV-infected DEFs were measured by ELISA. (B) LDH release from NDRV-infected DEFs. (C) Cell viability of DEFs after NDRV infection was measured by CCK-8 assay. (D) Representative bright-field and PI staining images of DEFs after NDRV infection. Scale bar, 30 μm. (E) WB analysis of GSDME, IL-1β, NLRP3, CASP-1, and σC in NDRV-infected DEFs at the indicated time points. Tubulin was used as the loading control. Data are presented as mean ± SD. Statistical significance was determined by ANOVA followed by Tukey’s post-hoc test. **p < 0.01, ***p < 0.001, ****p < 0.0001. (TIF) [file ppat.1014392.s004.tif]

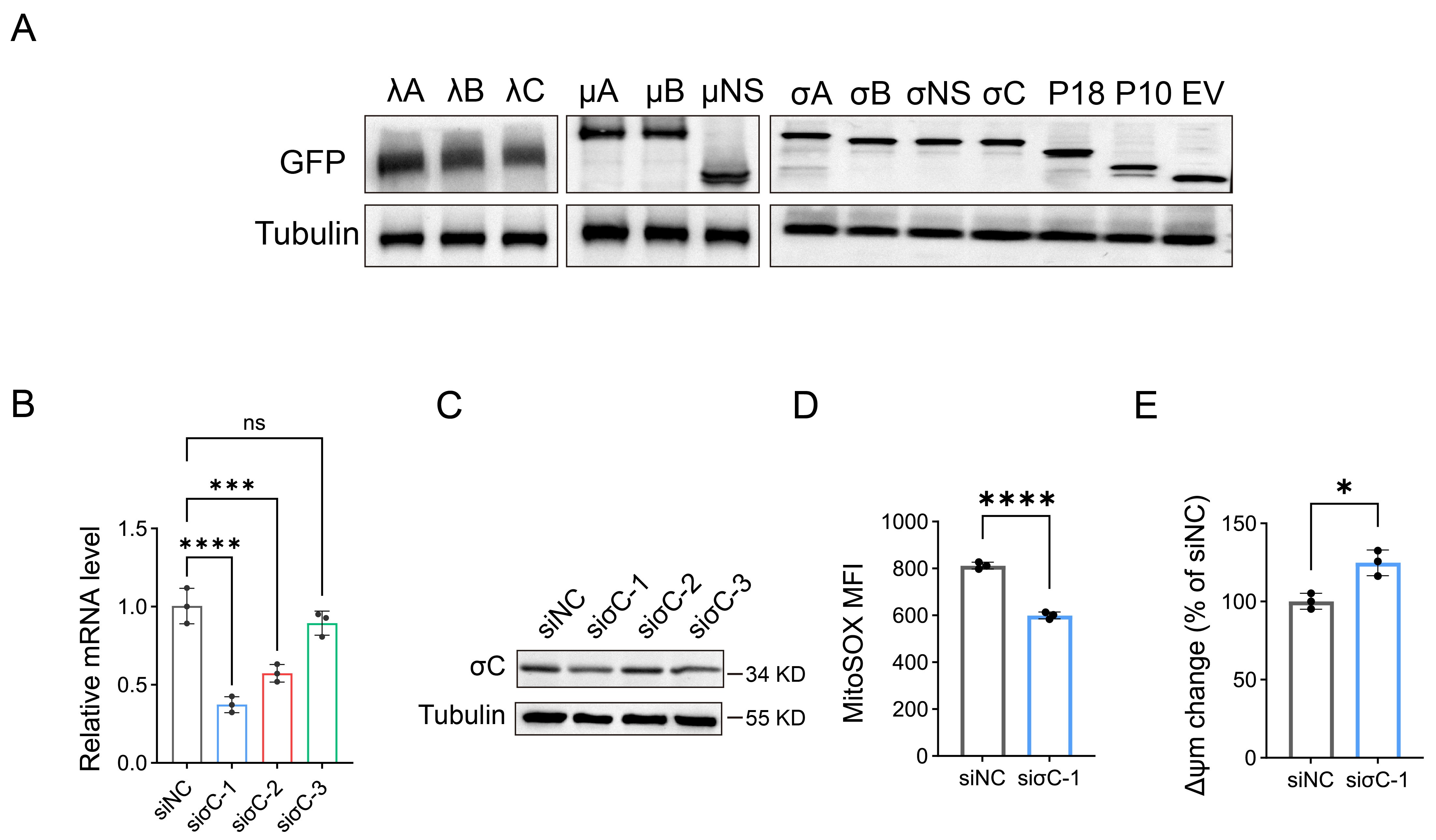

Supplement: S5 Fig — (A) WB validation of the expression of the 12 overexpressed NDRV proteins in DEFs. Tubulin was used as the loading control. (B) RT-qPCR validation of σC knockdown efficiency in DEFs transfected with three independent σC-targeting siRNAs. (C) WB validation of σC protein levels in DEFs transfected with three independent σC-targeting siRNAs. Tubulin was used as the loading control. (D–E) DEFs were transfected with siσC-1, with siNC as the negative control, and then infected with NDRV. MitoSOX Red MFI was quantified to assess mtROS accumulation (D), and ΔΨm changes were measured using the TMRM probe (E). Data are presented as mean ± SD. Statistical significance was determined by ANOVA followed by Tukey’s post-hoc test for multiple-group comparisons or unpaired Student’s t-test for two-group comparisons. ns, not significant; *p < 0.05, ***p < 0.001, **p < 0.0001. (TIF) [file ppat.1014392.s005.tif]

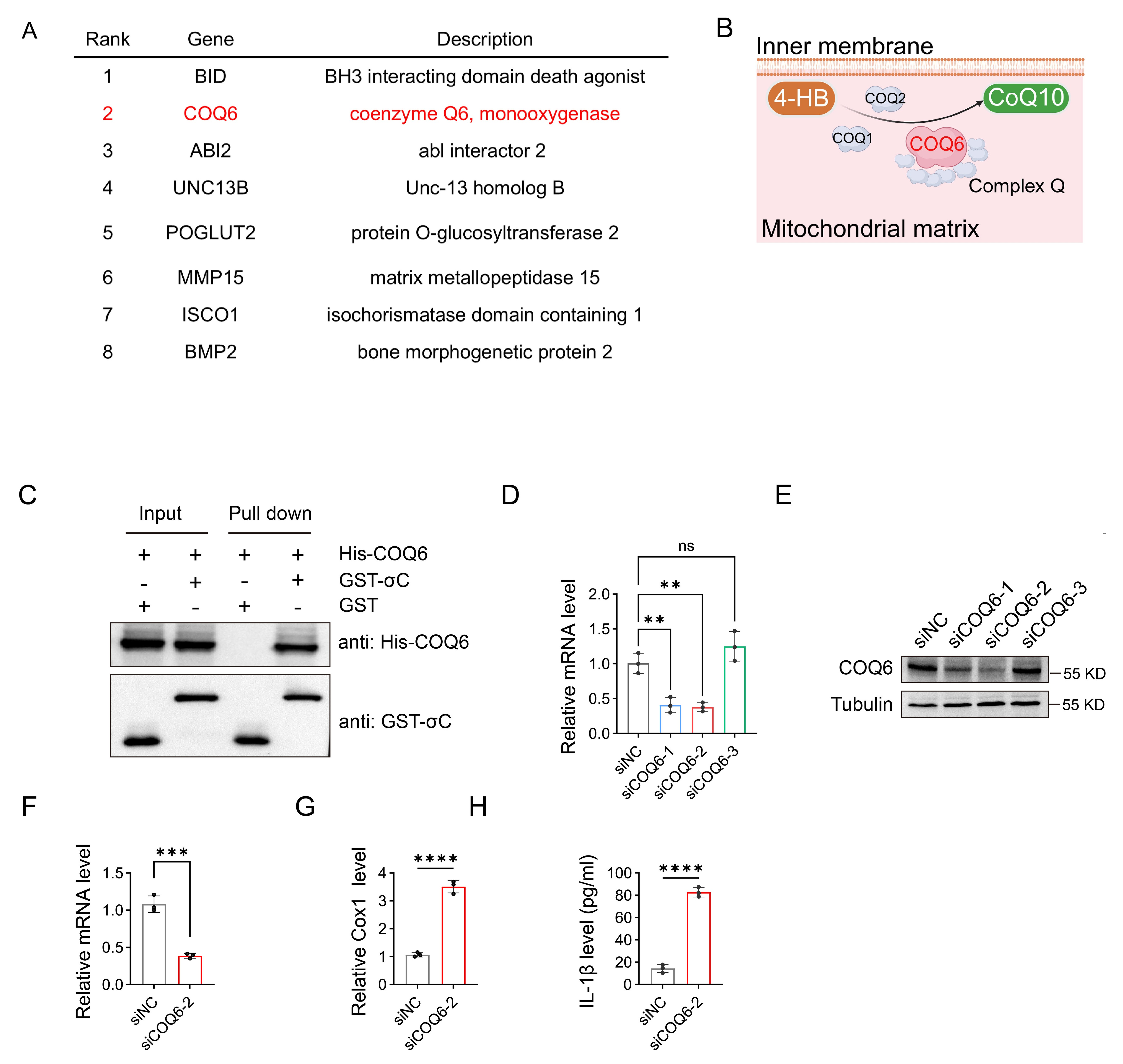

Supplement: S6 Fig — (A) Top candidate host proteins identified by IP–MS as potential σC-interacting factors. COQ6 is highlighted in red. (B) Schematic illustration of the role of COQ6 in the CoQ10 biosynthetic pathway and mitochondrial redox homeostasis. Panel B was created in BioRender. Yan, H. (2026) https://BioRender.com/q0yte9c. (C) Direct interaction between GST-σC and His-COQ6 was validated by GST pull-down assay, followed by WB detection with anti-His and anti-GST antibodies. (D) RT-qPCR validation of COQ6 knockdown efficiency in DEFs transfected with three independent COQ6-targeting siRNAs. (E) WB validation of COQ6 protein levels in DEFs transfected with three independent COQ6-targeting siRNAs. Tubulin was used as the loading control. (F) RT-qPCR validation of COQ6 mRNA levels in DEFs transfected with siCOQ6–2. (G) Cytosolic mtDNA levels in COQ6-knockdown DEFs were measured by qPCR using Cox1 primers. (H) IL-1β concentrations in culture supernatants from COQ6-knockdown DEFs were measured by ELISA. Data are presented as mean ± SD. Statistical significance was determined by ANOVA followed by Tukey’s post-hoc test for multiple-group comparisons or unpaired Student’s t-test for two-group comparisons. ns, not significant; **p < 0.01, ***p < 0.001, ***p < 0.0001. (TIF) [file ppat.1014392.s006.tif]

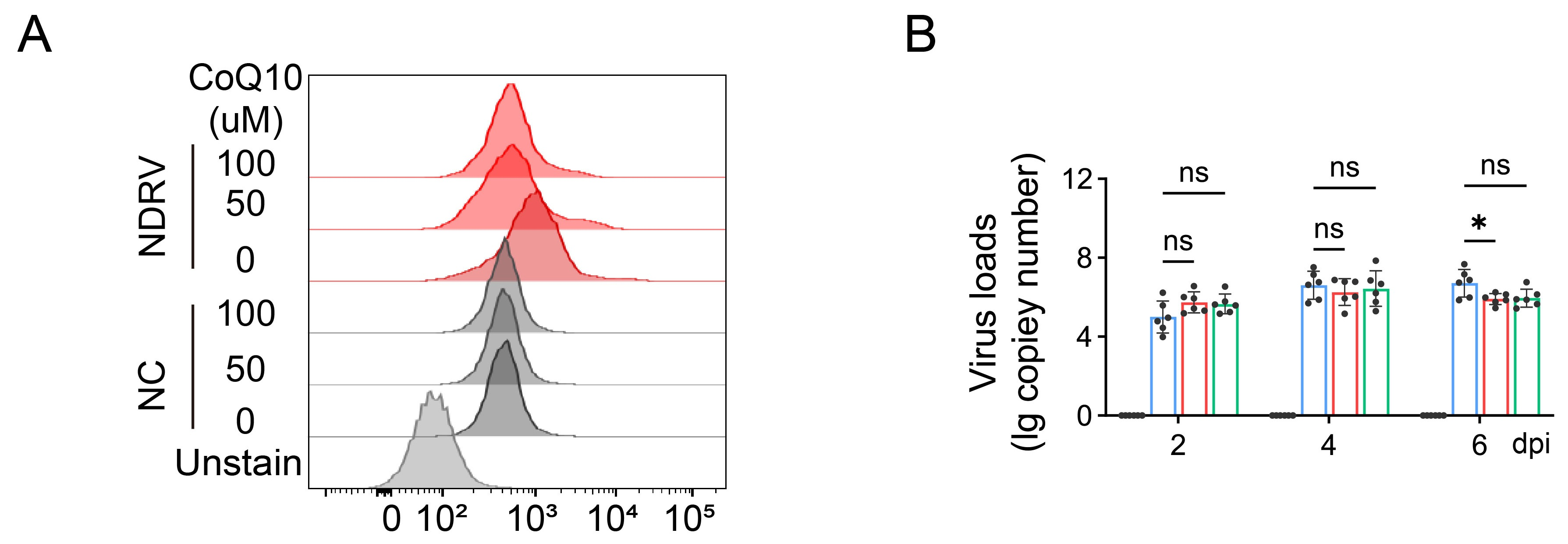

Supplement: S7 Fig — (A) PBMCs were mock-treated or infected with NDRV and then treated with CoQ10 (50 or 100 μM). Representative flow cytometry histograms of MitoSOX Red staining. (B) Viral loads in spleen homogenates from different treatment groups at the indicated dpi were determined by qPCR. Data are presented as mean ± SD. Statistical significance was determined by ANOVA followed by Tukey’s post-hoc test. ns, not significant; *p < 0.05. (TIF) [file ppat.1014392.s007.tif]

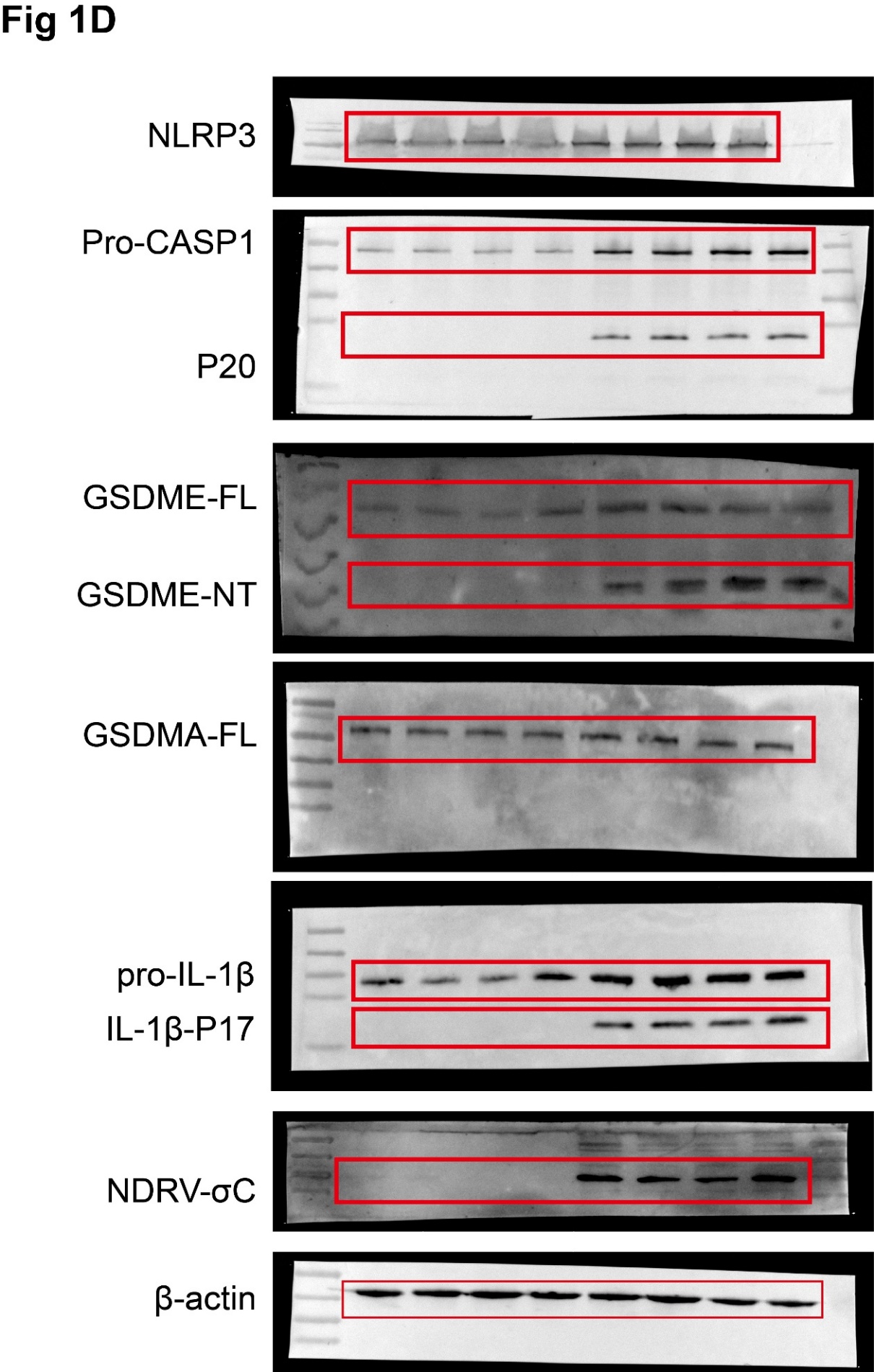


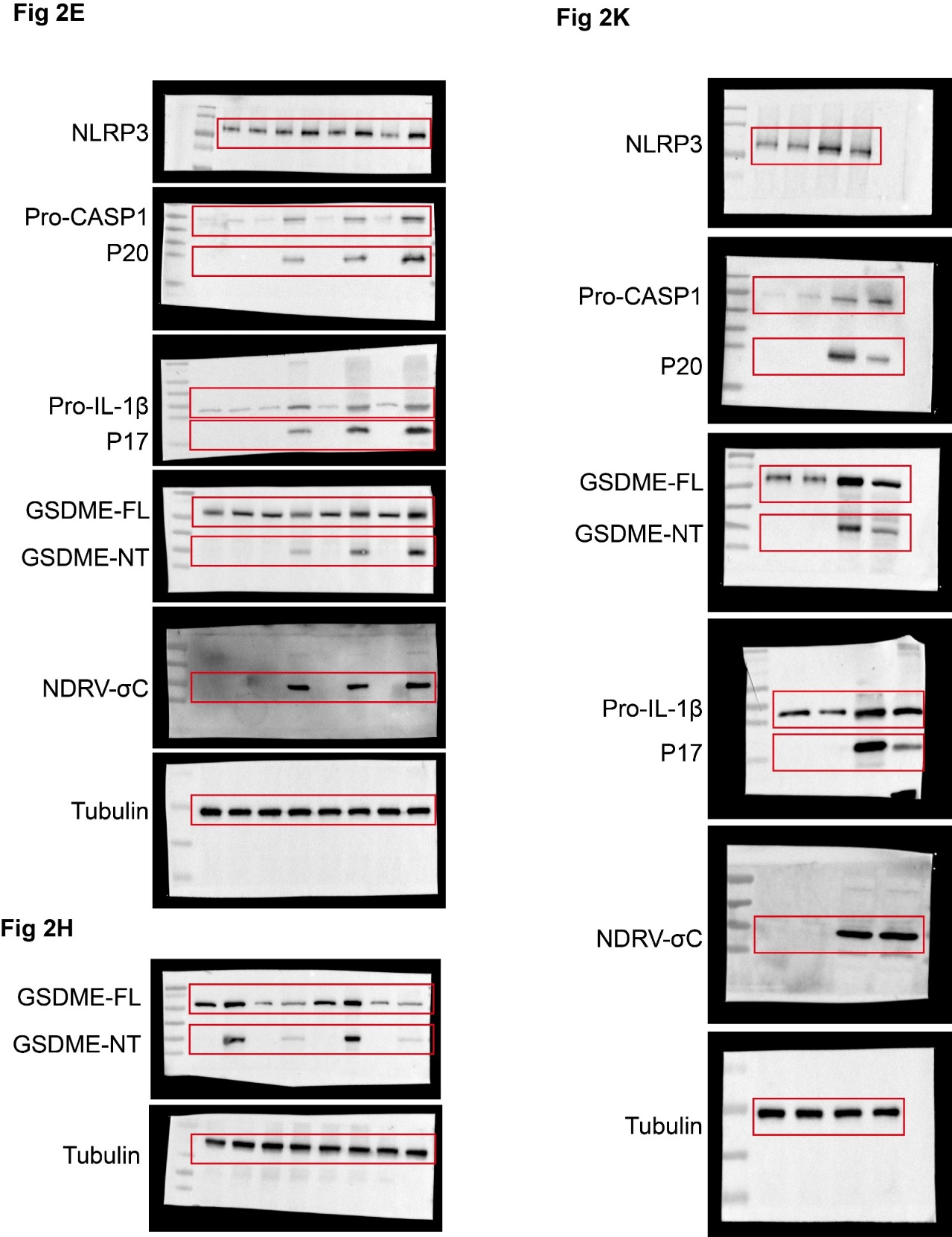


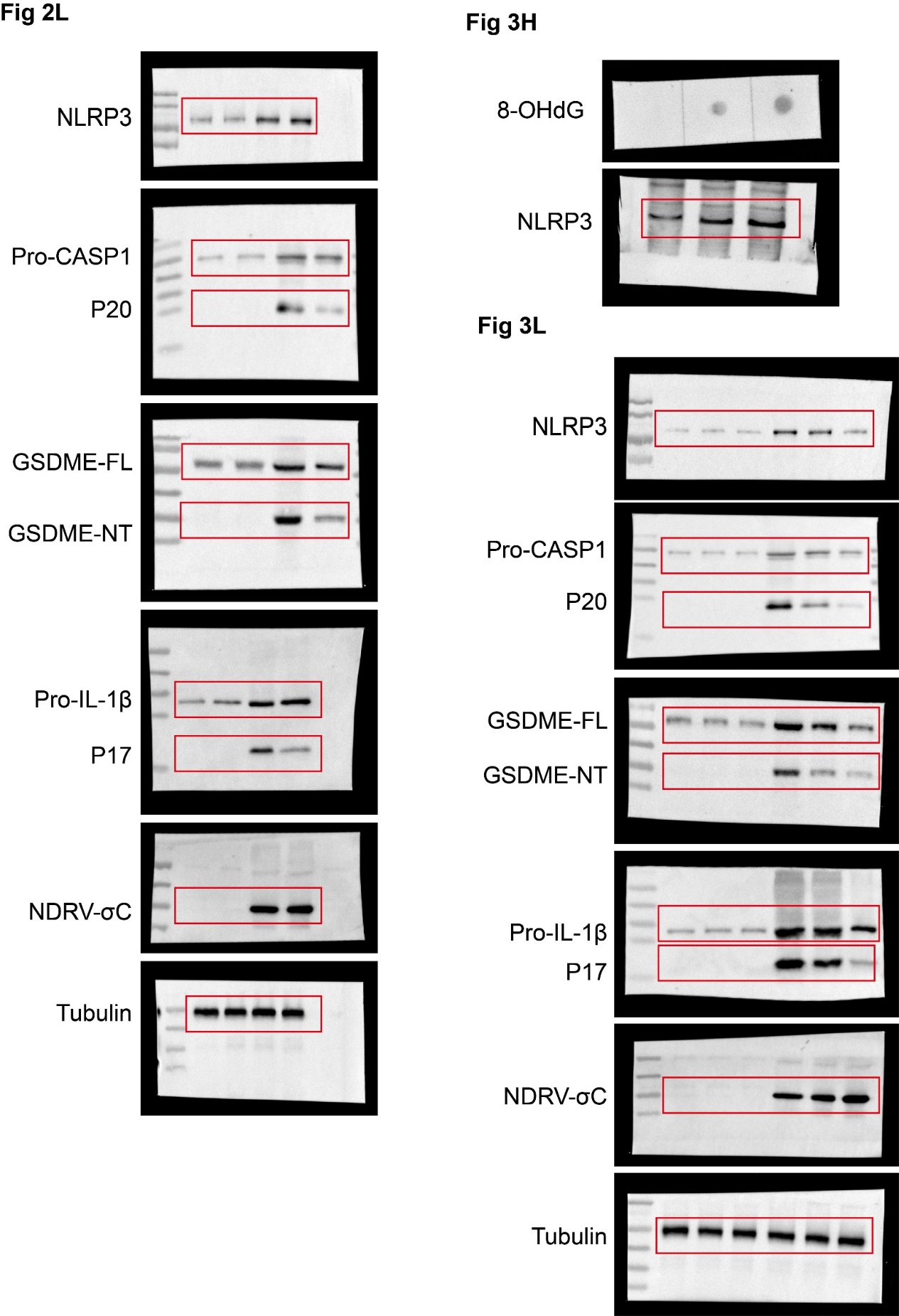


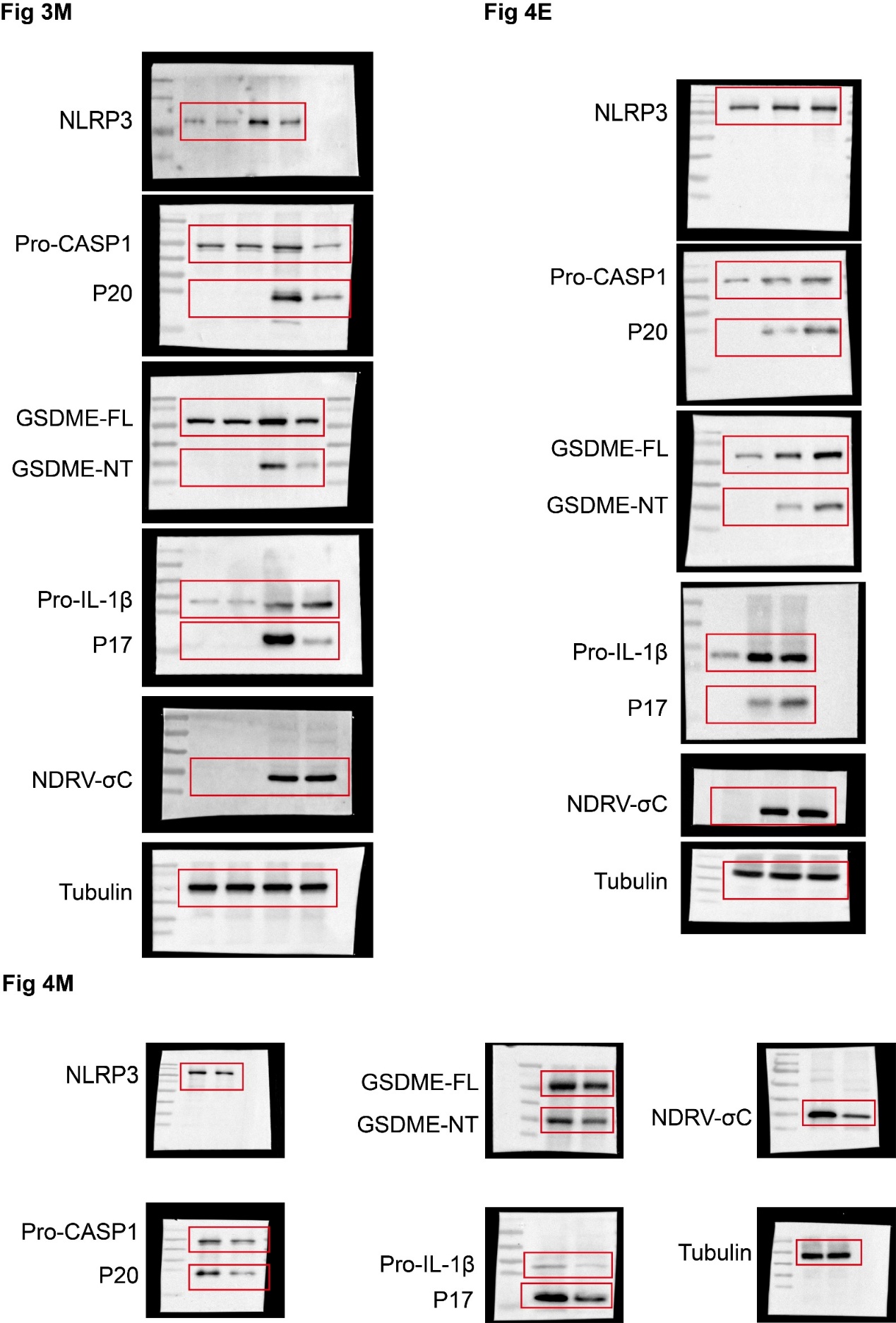

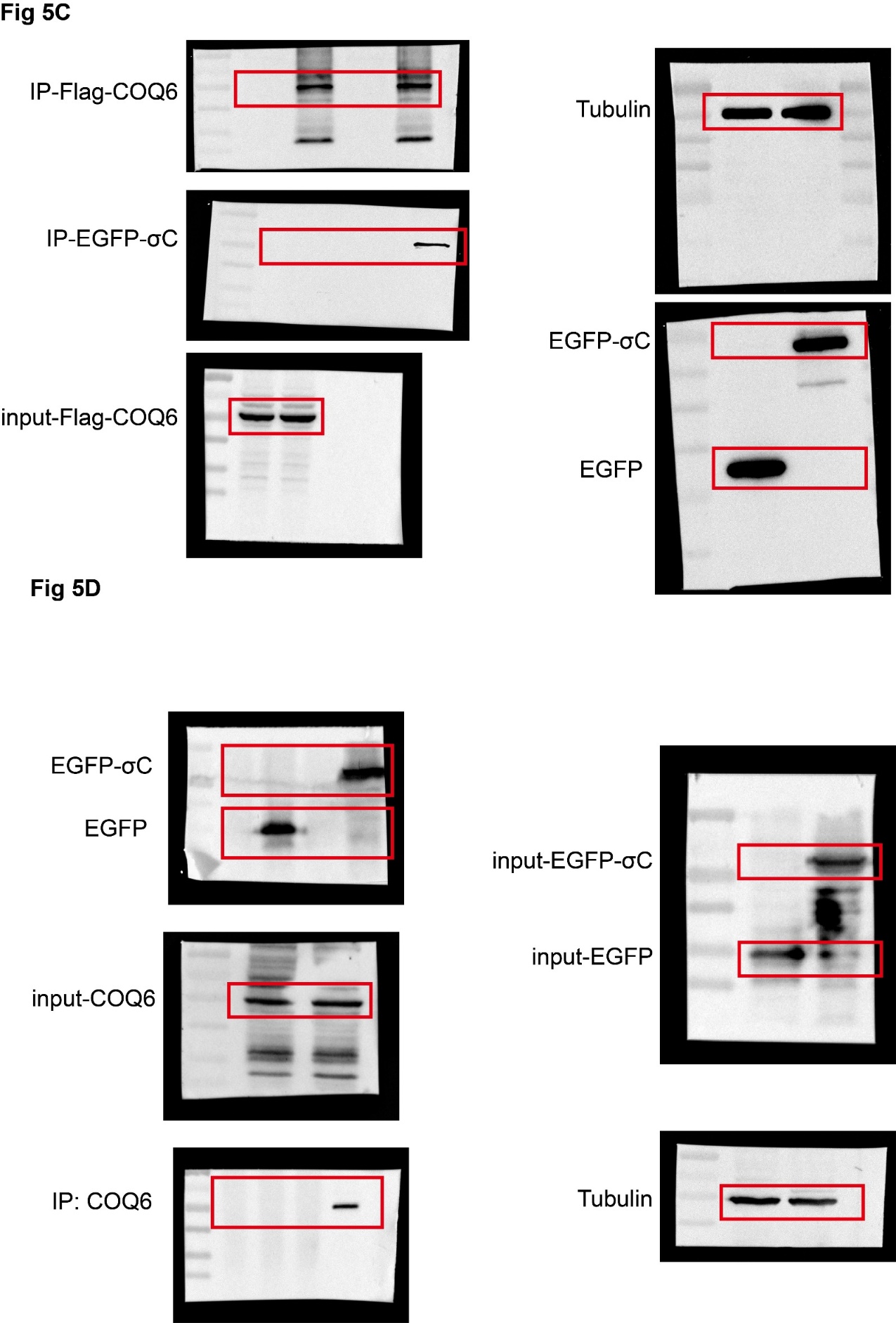

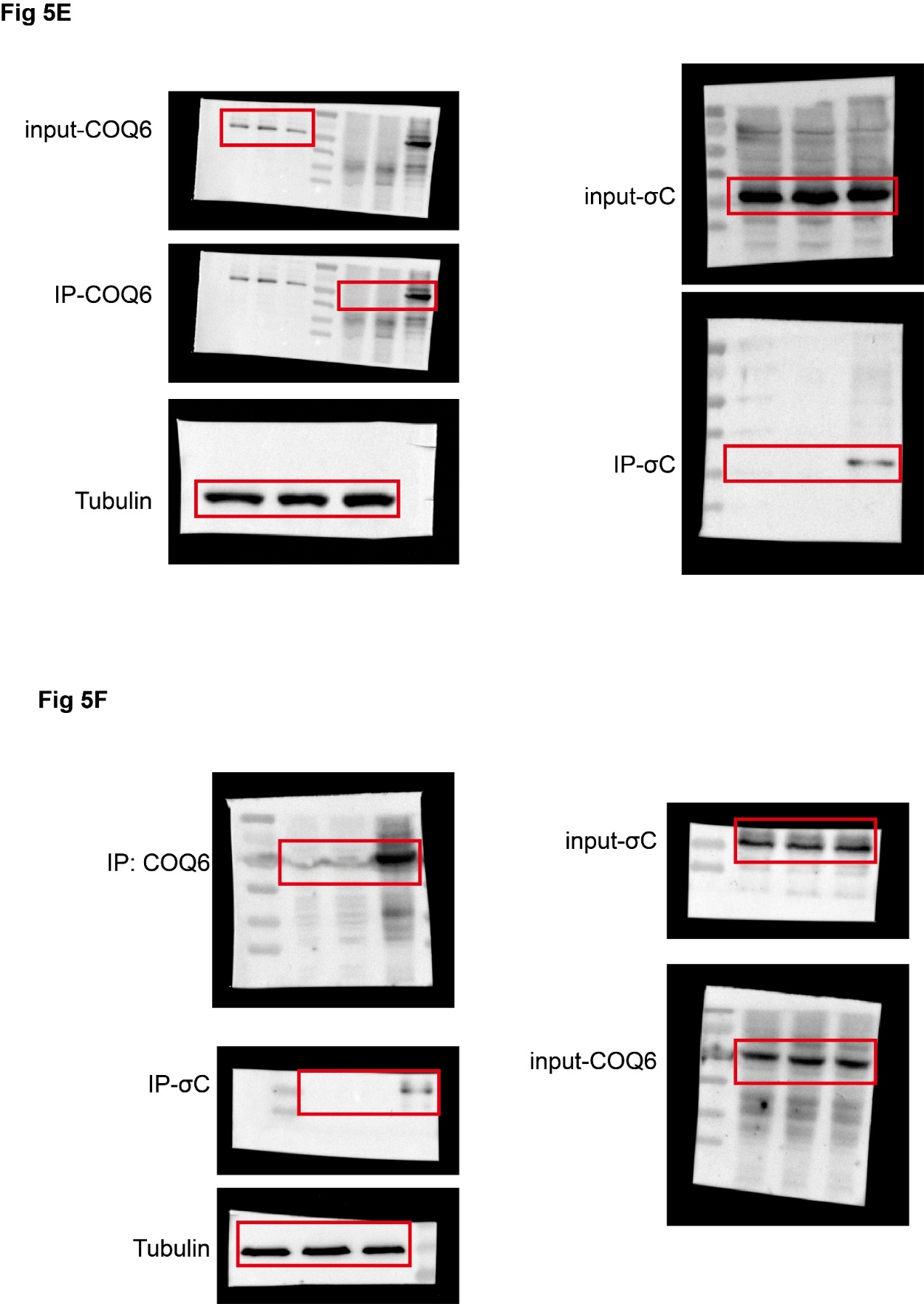

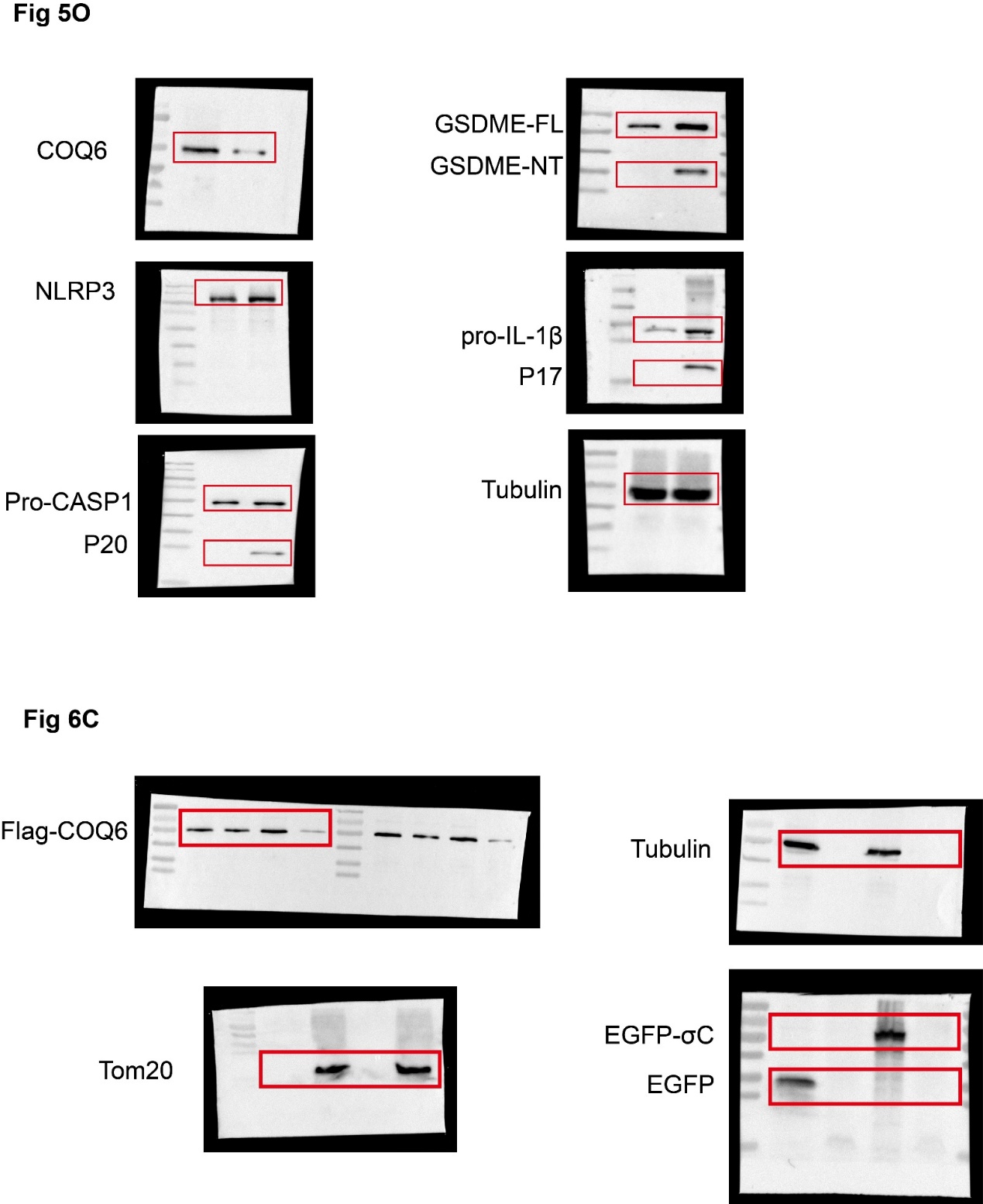

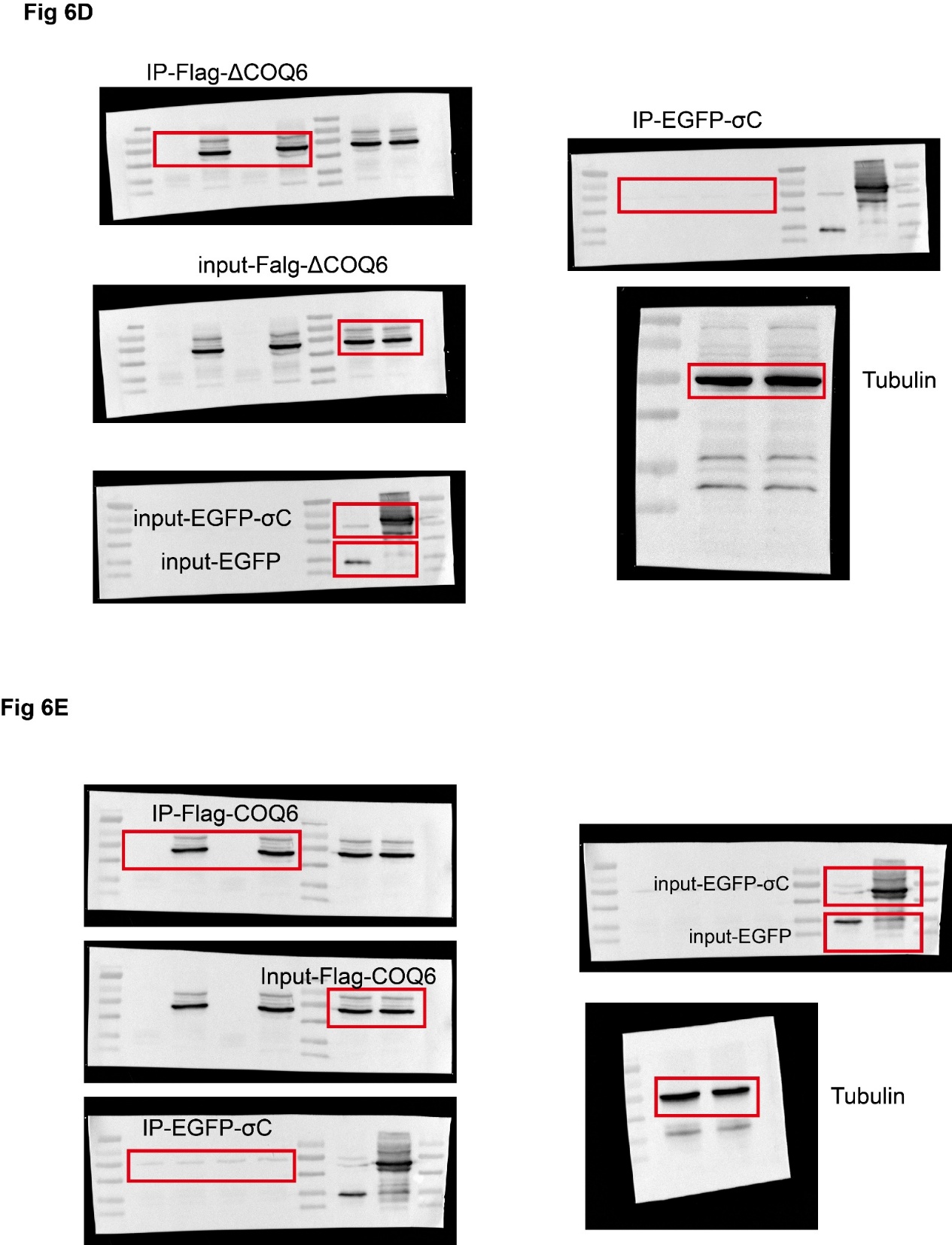

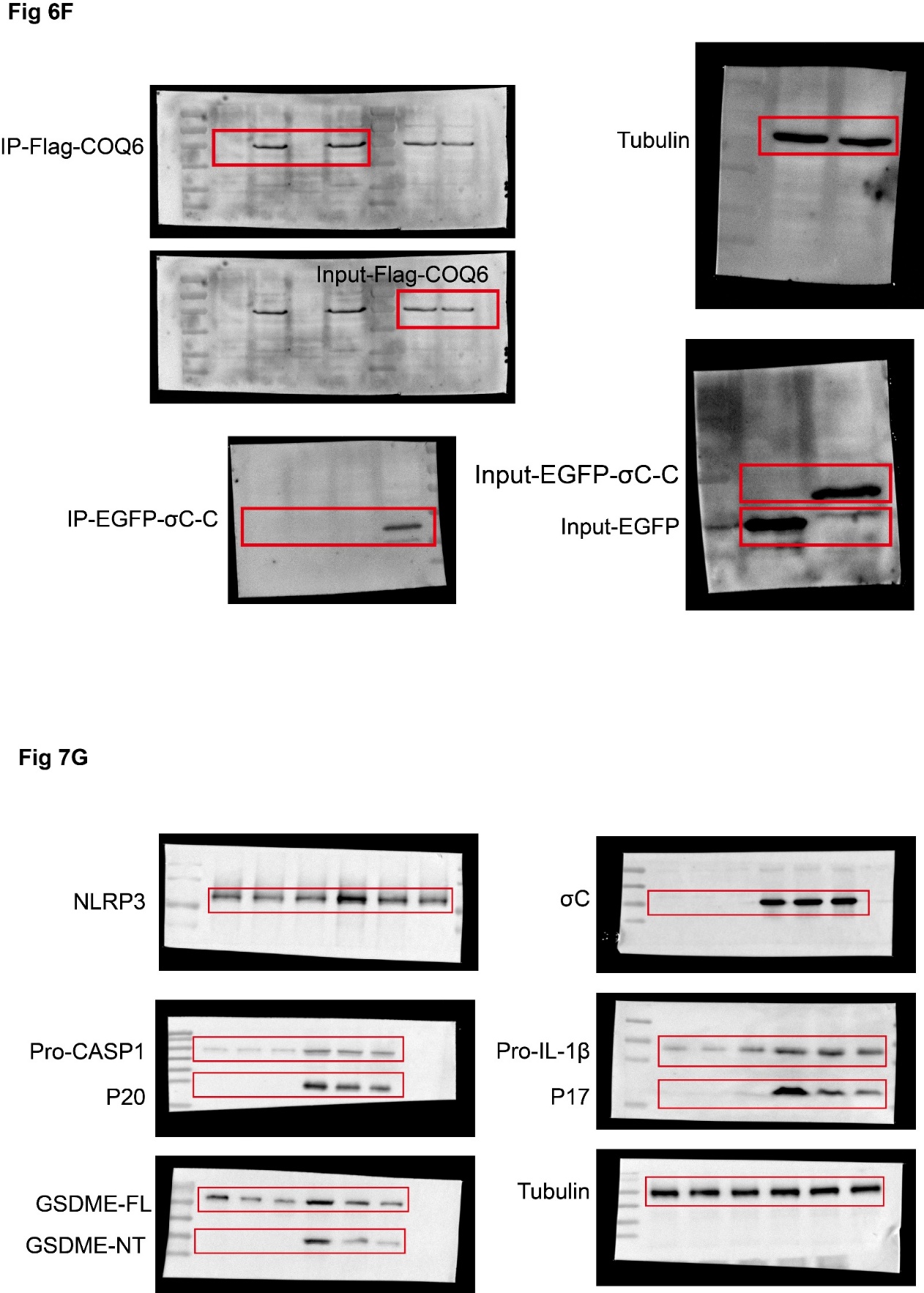


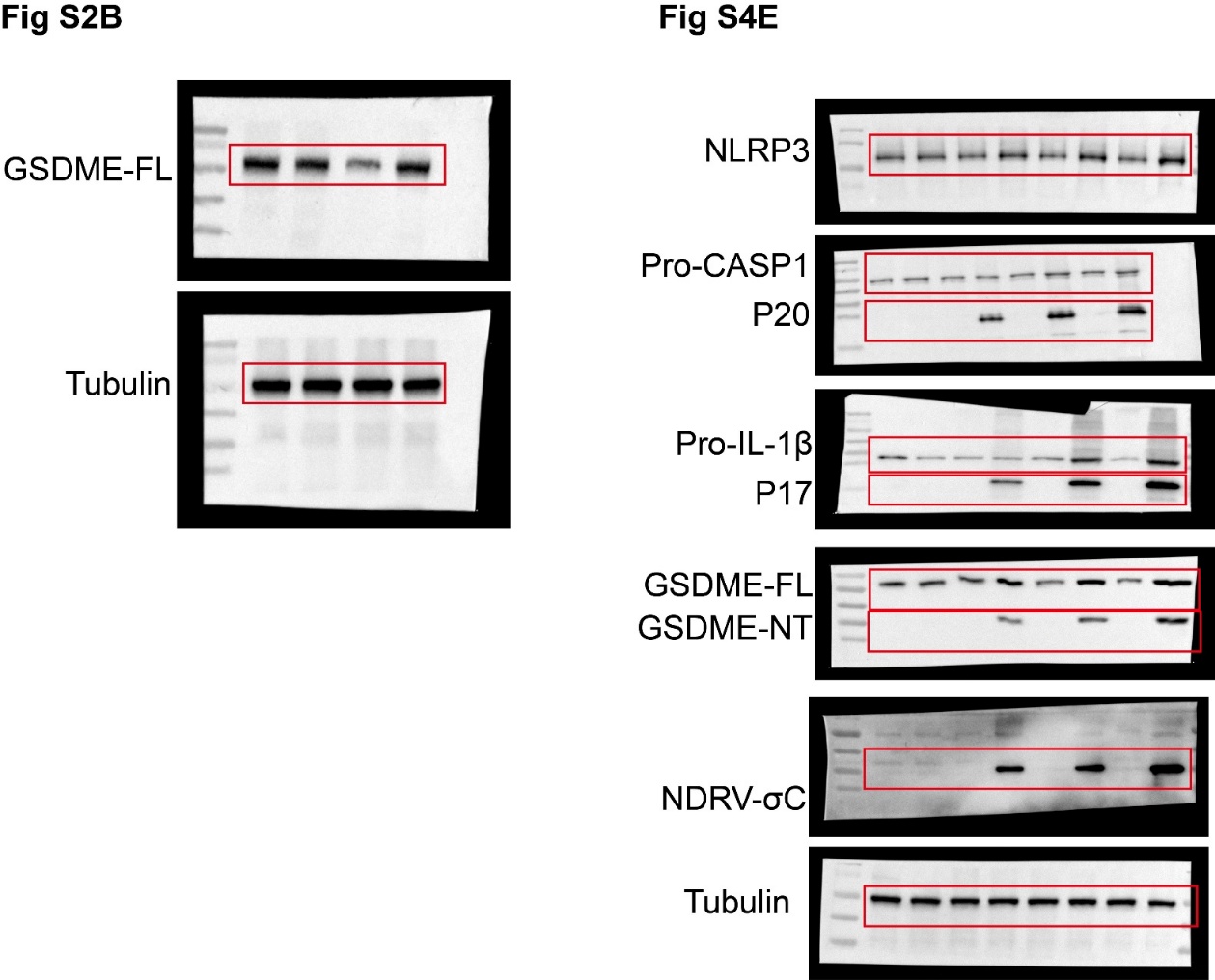

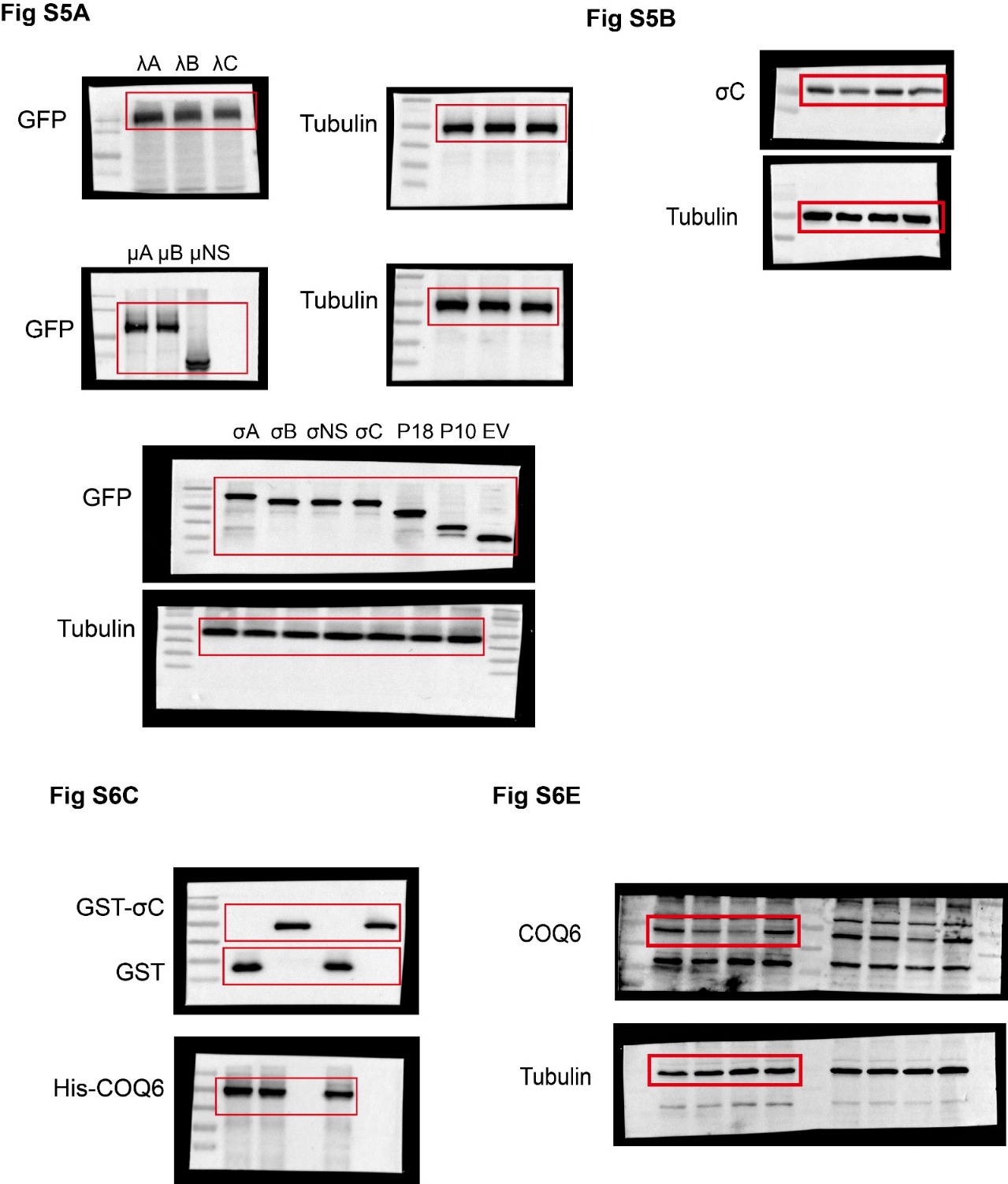

Supplement: S1 Raw Gel — (DOCX) [file ppat.1014392.s013.docx]
