# Supplementary material for: Novel duck reovirus σC hijacks the mitochondrial COQ6–CoQ10 axis to drive NLRP3-dependent pyroptosis
Source: PLoS Pathog. 2026 Jul 7;22(7):e1014392. doi: 10.1371/journal.ppat.1014392 (PMC13367899; doi:10.1371/journal.ppat.1014392)
Supplement: S1 Table — (DOCX) [file ppat.1014392.s008.docx]

| Gene | Primer (5’ to 3’) | Accession No. |
| --- | --- | --- |
| NDRV-λA | F: tacaagtactcagatctcgagATGAGTTCGCGCAAAGTGGC  R: gtaccgtcgactgcagaattcTTAGACTCTCACCAGACCGTAT | OQ704001.1 |
| NDRV-λB | F: tacaagtactcagatctcgagATGCATGTCAATGGGTTTGATG  R: gtaccgtcgactgcagaattcCTACCTCGTCTCAGCCCGC | OQ704002.1 |
| NDRV-λC | F: tacaagtactcagatctcgagATGGCTCAGATTAGAGGCCTTCG  R: gtaccgtcgactgcagaattcTTAGACTCTCACCAGACCGTATC | OQ704003.1 |
| NDRV-μA | F: tacaagtactcagatctcgagATGGCCTATCTAGCCACACCG  R: gtaccgtcgactgcagaattcTCAGTTCTCGTTTCCTACCGTGG | OQ704004.1 |
| NDRV-μB | F: tacaagtactcagatctcgagATGGGTAACGCGACGTCTGT  R: gtaccgtcgactgcagaattcCTATGGTTTGAACAACGTCTGT | OQ704005.1 |
| NDRV-μNS | F: tacaagtactcagatctcgagATGTCGTCAACCAAGTGGGG  R: gtaccgtcgactgcagaattcTCACAGGTCGTCCACCAGGT | OQ704006.1 |
| NDRV-σA | F: tacaagtactcagatctcgagATGGCGCGTGCCGTGTAC  R: gtaccgtcgactgcagaattcCTAGACAGTAAAAGTGGCTAGT | OQ704008.1 |
| NDRV-σB | F: tacaagtactcagatctcgagATGGAGGTGCGTGTGCCA  R: gtaccgtcgactgcagaattcTTACCACCTACACTCCAGGAAG | OQ704009.1 |
| NDRV-σC | F: tacaagtactcagatctcgagATGGATCGCAACGAGGTGAT  R: gtaccgtcgactgcagaattcCTAGCCCGTGGCGACGGT | OQ704007.1 |
| NDRV-σNS | F: tacaagtactcagatctcgagATGGACAACACCGTTCGTGTT  R: gtaccgtcgactgcagaattcCTACGCCATCCTAGCTGGAGAG | OQ704010.1 |
| NDRV-P18 | F: tacaagtactcagatctcgagATGTCACTCCCGCTAACCCC  R: gtaccgtcgactgcagaattcTCAGTTGTTGATTGTAGATCCGC | OQ704007.1 |
| NDRV-P10 | F: tacaagtactcagatctcgagATGGCTGACGGTGCATGC  R: gtaccgtcgactgcagaattcTTAAACGACTTGAACAGGATCG | OQ704007.1 |
| duCOQ6 | F: gatgacgacgataaggaattcATGGCCGCCATGGCGGTA  R: attaagatctgctagctcgagTCACTTGCTGGCAAAAGCCA | XM_038180795.2 |
| Du-ΔMTS-COQ6 | F: gatgacgacgataaggaattcTACGATGTGGTGGTGTCGGG  R: attaagatctgctagctcgagTCACTTGCTGGCAAAAGCCA | - |
| σC-C | F: tacaagtactcagatctcgagCGGATTCATTCATTTGGGCA  R: gtaccgtcgactgcagaattcCTAGCCCGTGGCGACGGT | - |
| σC-ΔC | F: tacaagtactcagatctcgagATGGATCGCAACGAGGTGAT  R: gtaccgtcgactgcagaattcCACGTTCATGGTTGCAGATGAC | - |

**S1 Table.** Primers used for constructing eukaryotic expression plasmids.
