# Supplementary material for: Novel duck reovirus σC hijacks the mitochondrial COQ6–CoQ10 axis to drive NLRP3-dependent pyroptosis
Source: PLoS Pathog. 2026 Jul 7;22(7):e1014392. doi: 10.1371/journal.ppat.1014392 (PMC13367899; doi:10.1371/journal.ppat.1014392)
Supplement: S2 Table — (DOCX) [file ppat.1014392.s009.docx]

**S2 Table.** siRNA sequences targeting σC, duck COQ6, and GSDME.

| siRNA | Strands | Sequence (5’ to 3’) |
| --- | --- | --- |
| σC-1 | Sense | CAAUCAACAACUGAAUUGUUG |
|  | Antisense | ACAAUUCAGUUGUUGAUUGUA |
| σC-2 | Sense | CUCUUUCUGUUCUUUGUCUCC |
|  | Antisense | AGACAAAGAACAGAAAGAGGG |
| σC-3 | Sense | GCUUUCAAAGACUCUAAGACA |
|  | Antisense | UCUUAGAGUCUUUGAAAGCUA |
| siCOQ6-1 | Sense | GGGUUACAUAGUGGAGAAUTT |
|  | Antisense | AUUCUCCACUAUGUAACCCTT |
| siCOQ6-2 | Sense | GGUCUACAUCCCAUGAACATT |
|  | Antisense | AUAUCACGAAAGCCCAGGUTT |
| siCOQ6-3 | Sense | GGUCUACAUCCCAUGAACATT |
|  | Antisense | UGUUCAUGGGAUGUAGACCTT |
| siGSDME-1 | Sense | GGUCUACAUCCCAUGAACATT |
|  | Antisense | AUAGUUCUGCAACAAUAGCTT |
| siGSDME-2 | Sense | CCCACUUGUUCUUUACGUUTT |
|  | Antisense | AACGUAAAGAACAAGUGGGTT |
| siGSDME-3 | Sense | GCAGAAGCCCAAGUAUCAUTT |
|  | Antisense | AUGAUACUUGGGCUUCUGCTT |
| siNC | Sense | UUCUCCGAACGUGUCACGUTT |
|  | Antisense | ACGUGACACGUUCGGAGAATT |
