# Supplementary material for: Novel duck reovirus σC hijacks the mitochondrial COQ6–CoQ10 axis to drive NLRP3-dependent pyroptosis
Source: PLoS Pathog. 2026 Jul 7;22(7):e1014392. doi: 10.1371/journal.ppat.1014392 (PMC13367899; doi:10.1371/journal.ppat.1014392)
Supplement: S3 Table — (DOCX) [file ppat.1014392.s010.docx]

| Target genes | Primer sequence (5’ to 3’) |
| --- | --- |
| D-Loop | F: TACGCATTGAGATGGTGGAAGTATTC  R: GCCTCTGGTTCCTCGGTCAG |
| COX1 | F: ATTAACTTCATTACCACAGCCATCAAC  R: GGAGTGATAGGAGGAGCAGGATG |
| EGFP | F: AGATCCGCCACAACATCGAG  R: TCTCGTTGGGGTCTTTGCTC |

**S3 Table.** Primers for mtDNA quantification.
