# Supplementary material for: Novel duck reovirus σC hijacks the mitochondrial COQ6–CoQ10 axis to drive NLRP3-dependent pyroptosis
Source: PLoS Pathog. 2026 Jul 7;22(7):e1014392. doi: 10.1371/journal.ppat.1014392 (PMC13367899; doi:10.1371/journal.ppat.1014392)
Supplement: S4 Table — (DOCX) [file ppat.1014392.s011.docx]

| Target genes | Primers sequence (5’ to 3’) |
| --- | --- |
| NLRP3 | F: GAAGACGATGATGGTCAGGAAGATG  R: ACTCACTTGCTTGGAAAGGGAAAG |
| ASC | F: ACGCACGCAGAAGCTCTAAT  R: ATGATGCTACCCTGTGCCTG |
| CASP-1 | F: TGACCTCTGACAGCACCTTCCTAG  R: GTGGTCTCATCTCTGCTCTTGGTTC |
| GSDME | F: CAGTGAGTGACAGTGGAAGTACAGTG  R: GAAGAGTTCAATCACACCATAGGCAATG |
| IL-1β | F: AGGCGGTAGAAGATGAAGCG  R: TGGGCATCAAGGGCTACAAG |
| β-actin | F: CTTTCTTGGGTATGGAGTCCTG  R: TGATTTTCATCGTGCTGGGT |

**S4 Table.** Primers used for RT-qPCR.
