# Supplementary material for: Novel duck reovirus σC hijacks the mitochondrial COQ6–CoQ10 axis to drive NLRP3-dependent pyroptosis
Source: PLoS Pathog. 2026 Jul 7;22(7):e1014392. doi: 10.1371/journal.ppat.1014392 (PMC13367899; doi:10.1371/journal.ppat.1014392)
Supplement: S5 Table — (DOCX) [file ppat.1014392.s012.docx]

**S5 Table.** Detailed information for the antibodies used in this study.

| Reagent | Dilution ration | Source | Catalog |
| --- | --- | --- | --- |
| IL-1β Rabbit Polyclonal Antibody | 1:1000 | MCE | HY-P80720 |
| NLRP3 Mouse Monoclonal antibody | 1:2000 | MCE | HY-P80246 |
| Caspase 1/P20 Rabbit Monoclonal Antibody | 1:1000 | Cell Signaling Technology | #4199 |
| GSDME Mouse Monoclonal antibody | 1:3000 | Proteintech | 68102-1-Ig |
| GSDMA Rabbit Polyclonal antibody | 1:2000 | Proteintech | 30354-1-AP |
| 8-OHdG Rabbit Polyclonal Antibody | 1:1000 | MCE | HY-P81140 |
| ASC Rabbit Polyclonal Antibody | 1:1000 | MCE | HY-P80548 |
| COQ6 Mouse Monoclonal antibody | 1:3000 | Proteintech | 67162-1-Ig |
| TOM20 Rabbit Polyclonal Antibody | 1:5000 | Proteintech | 11802-1AP |
| β-Tubulin | 1:5000 | Proteintech | 66240-1-Ig |
| β-actin Mouse Monoclonal antibody | 1:5000 | Engibody | AT0001 |
| GFP Mouse Monoclonal antibody | 1:5000 | Engibody | AT0055 |
| GFP Rabbit Monoclonal antibody | 1:5000 | Engibody | AT1598 |
| His Mouse Monoclonal antibody | 1:5000 | Engibody | AT0025 |
| GST Rabbit Monoclonal antibody | 1:5000 | Engibody | AT0043 |
| FlAG Rabbit Monoclonal antibody | 1:5000 | Engibody | AT0502 |
| FLAG Mouse Monoclonal antibody | 1:5000 | Engibody | AT0022 |
| Goat anti-Mouse IgG, Alexa Fluor™ 488 | 1:500 | Invitrogen | A-11001 |
| Goat anti-Rabbit IgG, Alexa Fluor™ 594 | 1:500 | Invitrogen | A-11012 |
| MMC950 | - | MCE | HY-12815 |
| EtBr | - | MCE | HY-D0021 |
| VX-765 | - | MCE | HY-13205 |
| Mito-TEMPO | - | MCE | HY-112879 |
| *E. coli* LPS | - | MCE | HY-D1056 |
| Nigericin | - | MCE | HY-127019 |
| Image-iT^TM^ TMRM Reagent | - | Invitrogen | I34361 |
| MitoSOX^TM^ Red Mitochondrial Superoxide Indicator | - | Invitrogen | M36008 |
